# Genetic structure and first genome‐wide insights into the adaptation of a wild relative of grapevine, *Vitis berlandieri*

**DOI:** 10.1111/eva.13566

**Published:** 2023-06-09

**Authors:** Louis Blois, Marina de Miguel, Pierre‐François Bert, Nabil Girollet, Nathalie Ollat, Bernadette Rubio, Vincent Segura, Kai P. Voss‐Fels, Joachim Schmid, Elisa Marguerit

**Affiliations:** ^1^ EGFV, Univ. Bordeaux, Bordeaux Sciences Agro, INRAE, ISVV Villenave d'Ornon France; ^2^ Department of Grapevine Breeding Geisenheim University Geisenheim Germany; ^3^ AGAP Institut, Univ Montpellier, CIRAD, INRAE, Institut Agro Montpellier France

**Keywords:** genome‐wide association, genotyping by sequencing, grapevine, long reads, population genetics, rootstock, whole‐genome sequencing

## Abstract

In grafted plants, such as grapevine, increasing the diversity of rootstocks available to growers is an ideal strategy for helping plants to adapt to climate change. The rootstocks used for grapevine are hybrids of various American *Vitis*, including *V*. *berlandieri*. The rootstocks currently use in vineyards are derived from breeding programs involving very small numbers of parental individuals. We investigated the structure of a natural population of *V*. *berlandieri* and the association of genetic diversity with environmental variables. In this study, we collected seeds from 78 wild *V*. *berlandieri* plants in Texas after open fertilization. We genotyped 286 individuals to describe the structure of the population, and environmental information collected at the sampling site made it possible to perform genome–environment association analysis (GEA). De novo *long‐read whole‐genome sequencing was performed on V*. *berlandieri* and a STRUCTURE analysis was performed. We identified and filtered 104,378 SNPs. We found that there were two subpopulations associated with differences in elevation, temperature, and rainfall between sampling sites. GEA identified three QTL for elevation and 15 QTL for PCA coordinates based on environmental parameter variability. This original study is the first GEA study to be performed on a population of grapevines sampled in natural conditions. Our results shed new light on rootstock genetics and could open up possibilities for introducing greater diversity into genetic improvement programs for grapevine rootstocks.

## 1 INTRODUCTION

In the context of climate change, the resilience of plants in natural populations and the productivity of agronomic species are compromised (Parmesan & Yohe, 2003; Root et al., 2003). The increasing frequency and intensity of threats such as drought, nutrient shortages, and the arrival of new pests and pathogens are introducing new challenges into plant breeding programs, which are increasingly called upon to develop new varieties able to overcome these environmental pressures.

Natural selection has tested a much wider range of gene combinations under various environmental pressures than could ever be tested in plant breeding programs (Cortés & López‐Hernández, 2021). For this reason, studies of the genetic basis of the adaptation of wild relatives of cultivated species to their native environments can provide useful genetic potential for incorporation into breeding programs (Condon et al., 2004; Vadez et al., 2014).

In recent years, improvements in sequencing technologies have made deeper explorations of the genetic basis of phenotype variability possible. Genome‐wide quantitative genetic studies can identify markers associated with traits of interest. Following their identification and validation, these quantitative trait loci (QTL) can be used in marker‐assisted selection (MAS; Tuberosa et al., 2007) to improve agronomic traits with a simple genetic architecture in crop plants; this approach has been used to increase yield in maize, rice, barley, and soybean (Francia et al., 2005). Genome‐wide association studies (GWAS) are based on the combination of ancestral recombination events (Nordborg & Tavaré, 2002) and information about linkage disequilibrium (LD); they can be used to detect associations between allelic and phenotypic variation. This approach makes it possible to hone in on the positions of loci controlling traits of interest by making use of the large number of recombination events that have occurred over many generations in natural populations. The main limitation of GWAS has been the extent of LD, which can be affected by many factors, including structuring of the population, population size, and genetic drift (Remington et al., 2001), leading to high false discovery rates and inconsistent results. Population structure and kinship are two major confounding factors in the detection of genotype–phenotype associations (Kang et al., 2008, 2010; Santure & Garant, 2018; Zhang et al., 2010). However, it is possible to control for the false discovery rate in current statistical models without increasing computing time by considering kinship, structure, and LD (Huang et al., 2018).

A particular case of GWAS uses environmental variables instead of phenotypes to identify a link between genetics and environment (genome–environment association, GEA) in wild populations (Santure & Garant, 2018). This makes it possible to explore the genetic basis of adaptation to the environment, and to identify loci with allele frequencies correlated with climatic data (Bragg et al., 2015). GEA studies are complementary to GWAS, as they can reveal adaptive patterns that are difficult to detect with GWAS approaches, and can identify the major environmental forces behind natural selection (Rellstab et al., 2015). Approaches of this type have been applied to the wild relatives of several crops, including barley (Abebe et al., 2015), as a means of identifying putative adaptive loci and selecting gene pools adapted to specific environmental conditions. In perennial species, such as trees, GEA has been successfully used to identify populations displaying potential preadaptation to the predicted future climate (De La Torre et al., 2019; Pluess et al., 2016). It could ultimately be used in the development of genetic markers to assist breeding strategies and to facilitate the precise selection of new wild genotypes for inclusion in breeding programs (Cortés et al., 2022).

The genetic load of breeding populations has also been identified as one of the main challenges in the transition to next‐generation breeding (Wallace et al., 2018). According to population genetics theory, most of the new mutations occurring in a population are neutral or slightly deleterious (Kimura, 1983; Ohta, 1973). Mutations with a strong deleterious effect should be rapidly eliminated by purifying selection. However, the efficacy of purifying selection for removing harmful alleles may be compromised in certain situations, such as demographic bottlenecks (González‐Martínez et al., 2017; Peischl et al., 2013), and in the presence of Hill–Robertson interference (i.e., a phenomenon that links alleles with potentially different fitness values in regions of low recombination; Hill & Robertson, 1966). Decreases in the efficacy of purifying selection lead to the accumulation of deleterious mutations that may compromise the fitness of natural populations or the productivity and resilience of crop species. The identification, control, and repair of such mutations in major crop species is, therefore, crucial for the persistence of natural populations and for breeding programs.

Grapevine (*Vitis vinifera*) breeding programs are particularly challenging because this perennial plant species has been grafted onto rootstocks since the phylloxera crisis in the nineteenth century. *Vitis vinifera* is used as the scion, to maintain grape yield and quality, but other *Vitis* species resistant to phylloxera are used as rootstocks. These rootstocks are derived from hybridizations between American species (mostly *V*. *riparia*, *V*. *rupestris*, and *V*. *berlandieri*) and sometimes with *V*. *vinifera* (Galet, 1988). Their selection for use in vineyards is based on their tolerance to phylloxera, chlorosis and water‐deficit tolerance, and the vigor conferred. Several studies have investigated genetic structure in grapevine (Aradhya et al., 2003; Arroyo‐García et al., 2006; Cipriani et al., 2010; Frenkel et al., 2012; Grassi et al., 2003; Imazio et al., 2006; Laucou et al., 2011, 2018; Myles et al., 2010, 2011; Péros et al., 2011, 2015, 2021). Rootstock identification is traditionally based on ampelographic traits (Galet, 1956; Ravaz, 1902) and genetic studies have essentially been restricted to *V. vinifera*, with little effort devoted to grapevine rootstock genetics (Arroyo‐García et al., 2006; Bianchi et al., 2020; de Andrés et al., 2007; Myles et al., 2011; Péros et al., 2011, 2015, 2021). New crosses are being performed to develop additional grapevine rootstocks, but mostly with the genotypes available in germplasm collections, and very little exploration of the genetic diversity existing in nature (Riaz et al., 2019). However, one recent study (Péros et al., 2021) based on SSR and SNP markers revealed a high level of genetic diversity in 421 genotypes of *V*. *aestivalis*, *V*. *cinerea* (var. *berlandieri* and *cinerea*), and *V*. *riparia*.

*Vitis berlandieri* is commonly used in crosses for the development of new grapevine rootstocks. Its hybrids perform well, but are difficult to use, mostly due to poor root emission after grafting (Boubals, 1966; Galet, 1988). However, the *V*. *berlandieri* genetic background is involved in hybridization to produce a number of widely used grapevine rootstocks, such as 110 Richter (*Vitis berlandieri* cv. Rességuier no. 2 × *Vitis rupestris* cv. Martin), Fercal (Berlandieri Colombard no. 1 B × 31 Richter), Gravesac (161‐49 Couderc × 3309 Couderc), and SO4 (*Vitis berlandieri* × *Vitis riparia*). The *V*. *berlandieri* genotypes used for hybrid creation were selected rapidly at the start of the twentieth century on the basis of surveys performed across the USA. Unfortunately, this genetic background has never been explored more deeply, and the diversity and genetic architecture of traits of interest in *V*. *berlandieri* in the wild remains unknown. This species is endemic to the Edwards Plateau area in Texas (USA), a dry, chalky region (Schmid et al., 2009). In a previous study on American genetic backgrounds, *V*. *berlandieri*, which is considered to belong to the *V*. *cinerea* subgroup, was clearly separated from other groups (Péros et al., 2021). A GEA approach was carried out on 130 Vitis accessions including 22 *V*. *berlandieri* accessions to explore the SNP associations between bioclimatic variables and bacterial levels after infection with *Xylella fastidiosa*, the causative agent of Pierce's disease (Aguirre‐Liguori et al., 2022; Morales‐Cruz et al., 2021). Moreover, whole‐genome sequencing studies were carried out with *V*. *riparia* (Girollet et al., 2019) and 21 Vitis accessions (Liang et al., 2019), but it remains a neglectable part of the grapevine studies more focused on *Vitis vinifera* diversity for scion‐related traits (Tello & Ibañez, 2023). Thereby, the genetic background of wild *Vitis* remain poorly explored.

In this study, we addressed the following objectives: (i) characterization of the genetic structure of *V*. *berlandieri*; we generated genome‐wide molecular markers from a de novo assembly of the *V*. *berlandieri* genome for this purpose; (ii) exploration of the genomic features of this species in terms of the extent of linkage disequilibrium and genetic diversity and their variation according to genetic structure; (iii) study of deleterious allele accumulation in the different subpopulations; and (iv) identification of genes potentially involved in the adaptation of this species to the environment.

## 2 MATERIALS AND METHODS

### 2.1 Plant material

In 2005, 78 wild female *V*. *berlandieri* plants were ampelographically identified in Edwards Plateau (transect of 40,000 km^2^; from N 31°23′ W 100°2′ to N 29°43′ W 97°26′) in Texas, USA. The coordinates and elevation of each sampling site were recorded. We harvested approximately 40,000 seeds from these plants after open fertilization, and about 5000 of these seeds were sown in a field at Geisenheim University, Germany. We selected 286 genotypes within this population on the basis of vigor, such that each initial “mother” plant was represented by four genotypes (half‐sibs) to encompass the available genetic diversity.

### 2.2 Reference genome

The reference genotype for this study was “*V*. *berlandieri* 10585” (NCBI, BioProject ID: PRJNA886625) from the collection of INRAE‐Bordeaux, (Villenave d'Ornon, France). This genotype was selected on the basis of its rooting capacity and use in other experiments. “*V*. *berlandieri* 10585” leaves were harvested from the INRAE Bordeaux grapevine collection (Villenave d'Ornon, France). Two young leaves with a width of about 5 cm were collected, frozen, and stored in a −80°C freezer for DNA extraction. DNA was extracted with the Tip 100 Qiagen Genomic kit, according to a slightly modified version of the manufacturer's protocol. We incubated 0.5 g of ground plant material with 9.5 mL of G2 buffer supplemented with 1% PVP‐40, 19 μL RNase A, and 500 μL proteinase K for 3 h at 50°C for lysis. The lysate was subjected to tip filtration and the DNA was precipitated with isopropanol, centrifuged for 15 min at 5000 *g*, washed with 70% ethanol, and resuspended in 50 μL TE buffer. The quality and molecular weight of the DNA isolated were checked. An A_260_/A_280_ ratio between 1.8 and 2.0 and an A_260_/A_230_ ratio between 2.0 and 2.2 were obtained, and an Agilent Genomic DNA Screentape analysis was performed. We used 10 μg of high‐quality DNA for sequencing. Samples were sequenced with Single‐Molecule Real‐Time PACBIO SEQUEL II HIFI long reads at the INRAE Clermont‐Ferrand GENTYANE platform (France).

DNA consensus call sequences obtained in BAM format were converted to Fastq format with the bam2fastq tool from the SMRTLink v11.0 PACBIO library. The HIFI sequencing DNA quality was checked with FastQC version 0.11.7. Paternal and maternal kmers were identified with yak‐0.1 software, using the parental reads. The outputs were then used in hifiasm‐v0.15.5 to bin long reads and to assemble the two haplotypes. For each haplotype, contigs were aligned with PN40024.v4 with minimap2 version 2.17. The best contig alignments were used to build an AGP file, and each pseudomolecule was then reconstructed. We refined the pseudomolecules by repeating the process, beginning with an alignment of each haplotype against the other, previously reconstructed haplotype. The embryophyta_odb10 lineage package from BUSCO 5.3.1 software was used in genome mode to estimate the completeness of all assemblies.

### 2.3 Genotyping by sequencing

Leaves were sampled from all 286 genotypes at Geisenheim University, Germany. Two leaf discs of 1.5 cm diameter were sampled from each genotype and placed on ice. The leaf discs were then frozen in liquid nitrogen and freeze‐dried in a Martin Christ, Beta 2‐8LD freeze‐dryer. DNA was extracted from all lyophilized samples as described by Cormier et al. (2019) in Corning/Costar deep 96‐well 1.1 mL plates. Libraries were prepared at UMR AGAP, CIRAD (Montpellier, France) as described by Elshire et al. (2011). Based on our results, the amount of DNA was normalized to 50 ng/mL. We prepared 96‐plex GBS banks with the restriction enzyme ApeKI. Illumina Hiseq 4000 short‐read 2 × 150 bp sequencing was performed by Genewiz. Three 96‐plex plates were used. The row data are available on NCBI, BioProject ID: PRJNA886619.

### 2.4 SNP calling

GBS data were processed with the Genotoul cluster, Toulouse, France. Reads were demultiplexed with a script available from https://github.com/timflutre (demultipley.py) and cleaned with Cutadapt (Martin, 2011) with filters “‐a AGATCGGAAGAGCGGTTCAGCAGGAATGCCGAG ‐A GAGATCGGAAGAGCGTCGTGTAGGGAAAGAGTGT ‐G CTCGGCATTCCTGCTGAACCGCTCTTCCGATCT ‐g ACACTCTTTCCCTACACGACGCTCTTCCGATCT ‐u 7 ‐U 7 ‐m 17.” Sequences were aligned with the *V*. *berlandieri* reference genome obtained in this study. VCFs were joined with GATK tools (McKenna et al., 2010), and 3,294,984 SNPs were obtained. SNPs were filtered, with the rejection of SNPs with a quality depth < 2.0, Fisher strand value >60.0, MQ < 40.0, MRankSum < −12.5, and ReadPosRankSum < − 8.0. These filters were applied one by one, as recommended in the GATK support documentation. In total, 3,294,747 SNPs were conserved and individuals with more than 80% missing data were filtered out (*n* = 281 genotypes retained). VCFtools (Danecek et al., 2011) was used for filtering based on the following criteria: minimum depth of 3, maximum of 50% missing data, minor allele frequency of 0.05, and a minimum mean depth of 5. In total, 104,378 SNPs were conserved. We considered 281 genotypes with less than 60% missing data.

### 2.5 Population structure

Two methods were used for the analysis of population structure. We first ran STRUCTURE v2.3.4 (Pritchard et al., 2000) on all 104,378 SNPs. The optimal number of subpopulations was determined as previously described (Evanno et al., 2005). One to 10 populations were allowed, with a burn‐in period of 20,000 and a Markov Chain‐Monte Carlo (MCMC) iteration number of 20,000 without prior knowledge of population affinities and three runs. The optimal number of populations was found to be two (*K* = 2). A new run was then performed, with a burn‐in period of 100,000 and 100,000 MCMC iterations. Genotypes with a membership proportion greater than 0.8 for a population were attributed to the population concerned. Genotypes not attributed to a particular population were considered to be admixed. The STRUCTURE results were then compared with those of a *k*‐means clustering method described elsewhere (Voss‐Fels et al., 2015). A genetic matrix distance was calculated with Roger's distance (RD). Clusters were identified by the unweighted pair‐group with arithmetic mean (UPGMA) method, based on Roger's genetic distance. Genotypes were then attributed to a cluster by the *k*‐means clustering method, with the Hartigan and Wong algorithm (1979). The optimal number of clusters was determined by plotting cluster numbers from 1 to 15 against the corresponding within‐cluster sum of squares (Voss‐Fels et al., 2015). This calculation was performed 20 times and the mean value of each run was reported. The optimal number of clusters was found, by eye, to be *K* = 5, as beyond this value, increasing the number of clusters did not significantly decrease the within‐cluster sum of squares. The results were visualized by plotting PCA results constructed with the first four principal components according to marker information. The number of clusters in the PCA was chosen based on the optimal *K* value from STRUCTURE analysis considering admixed individuals as a subpopulation (*K* = 3) and from the visualization of within‐cluster sums of squares (*K* = 5).

We tested for isolation by distance by calculating Nei's distance matrix between genotypes with the *adegenet* package in R. As there were several genotypes originating from each mother plant, the mean genetic distance was calculated between mother plants so as to obtain a single value for the genetic distance between two mother plants. A distance matrix was constructed from the GPS coordinates of the mother plants with the *sp* package in R. A Mantel correlation test was performed by the Spearman method, with 9999 permutations.

### 2.6 Linkage disequilibrium and genetic load

Linkage disequilibrium decay was estimated as the physical distance at which *r* ^2^ reached a value of 0.2, as previously described (Hill & Weir, 1988). Intrapopulation *F*_ST_ was calculated by STRUCTURE and interpopulation (*K* = 2) *F*_ST_ was calculated with VCFtools (‐‐weir‐fst‐pop option), using the default parameters. The *V*. *berlandieri* reference genome was annotated according to the Pinot noir reference genome (12X.v2; Canaguier et al., 2017), with liftoff1.6.1. In total, 39,250 of the 42,413 genes from the Pinot noir reference genome (92.5%) were found in *V*. *berlandieri*. The annotated *V*. *berlandieri* genome was added to the program as an additional reference genome. SnpEff (Cingolani et al., 2012) was run with default parameters, and two items of information were considered in our analyses: the predicted impact of the SNP on the DNA sequence and the effect of the SNP according to its location in the genome (see Table S2 for a detailed list of effects). Impacts were classified into four categories: “high” (e.g., loss of function of the protein), “moderate” (e.g., modification of protein efficacy), “low” (probably no impact on protein), and “modifier” (mostly noncoding variants or variants for which there was no evidence of impact). Kimura's neutral theory of evolution (Kimura, 1968) suggests that most mutations are deleterious or neutral. We therefore calculated the genetic load as follows:

(1) $$P_{\mathrm{del}}=\frac{\text{Number}\;\text{of}\;\text{minor}\;\text{alleles}\;\text{for}\;“\text{high}”\;\text{impact}\;\mathrm{SNP}}{\text{Number}\;\text{of}\;\text{alleles}\;\text{for}\;\mathrm{SNP}\;\text{with}\;“\text{moderate}”\;\text{and}\;“\text{high}”\;\text{impact}\times 2}$$

This Equation (1) considers “P_del_” as the proportion of deleterious alleles for one individual. SNPs with missing data were not considered for each individual. The accumulation of deleterious alleles in each population was evaluated as the mean P_del_ of the individuals assigned to each population according to STRUCTURE analysis divided by the number of deleterious alleles for all individuals from each population. The accumulation of deleterious alleles was compared between subpopulations by a Tukey test. For each SNP, snpEff could propose several impacts and effects. In such cases, we retained the first effect/impact proposed by the program, which was the most deleterious.

### 2.7 Genome‐wide association with environmental variables

Genome–environment association (GEA) analysis was performed with the elevation of the mother plants reported during sample and additional environmental information extracted from the TerraClimate platform (Abatzoglou et al., 2018) for each of the mother plant coordinates. The following environmental parameters were obtained for the 1991–2020 period, at a resolution of 4 km:

- Growing season temperature (GST, from April to October, Jones, 2006) and growing season temperature during the vegetative period (GST49, between April and September). The mean daily average temperature is calculated over the period concerned. This parameter affects earliness and grape quality (Jones, 2006).
- Growing season rainfall (GSR from April to October, Bois et al., 2017) and growing season rainfall during the vegetative period (GSR49, from April to September). This parameter is calculated as the cumulative amount of rainfall (mm) over the period concerned. It provides a rough estimate of the amount of water available to the plant during the corresponding period.
- Springtime rainfall (RRSPR), which is essentially GSR for the period between April and July. It can be used as an indicator of biotic pressure during the first few months of vegetative growth (Bois et al., 2017).
- Branas hydrothermal index (HYB; Branas et al., 1946), which evaluates the risk of grapevine exposure to disease.
- Winter freeze risk index (WFR; Bois et al., 2014) is the mean minimum temperature in January. If this temperature is >4°C, the risk is considered to be low, whereas the risk is considered high if it is <−11°C.
- Spring frost risk index (SFR; Bois et al., 2014) is the mean minimum temperature in April. The risk is considered low if this temperature is >12°C and high if it is <0°C.
- Heat stress index (HST; Bois et al., 2014) is the mean maximum temperature in July. The risk is considered to be low if this temperature is <25°C and high if it is >30°C.
- The Huglin index (HI; Tonietto & Carbonneau, 2004) provides information about the climate of the region. It combines mean air temperature (*T*, °C), the maximum air temperature (*T*_*x*_, °C), and day length coefficient (*d*) according to latitude, between April and September. This parameter may reflect the sugar‐producing potential of the plant during the vegetative period in a given context.

$$\mathrm{Hi}=\sum_{01/04}^{30/09}\frac{\left(T-10\right)+\left(T_{x}-10\right)}{2}d$$

- Cool night index (CI, Tonietto & Carbonneau, 2004) is the mean daily minimum air temperature in September. It provides qualitative information about the potential of wine‐producing regions based principally on the production of secondary metabolites (polyphenols, aromas).
- Dryness index (DI, Tonietto & Carbonneau, 2004) is an indicator of potential soil water availability according to the dryness of a climatic region. It is adapted from the soil index of Riou (Riou et al., 1994) and it affects ripening and wine quality (Carbonneau, 1998).

The Pearson coefficients of correlation between these environmental variables were explored. GST and GST49 were highly correlated, as were GSR and GSR49 (coefficients of 0.99 for both). We therefore included only GST49 and GSR49 in the PCA calculation. A GEA analysis was performed for elevation, and then for the first two principal components of the PCA performed with TerraClimate environmental parameters. Elevation was analyzed separately from the other environmental parameters because it was measured during the sampling campaign, whereas the other environmental variables were obtained by climatic interpolation.

We performed GEA with the BLINK model in GAPIT with default settings. Before running GWAS, the SNPs dataset has been imputed with the snp.impute function available in GAPIT using the “Major” argument in order to impute missing data by the major allele of each SNP. Because the imputation procedure led to changes in the allele frequency at each position, mainly for SNPs with a large number of missing data, a new MAF > 0.05 filtration was applied excluding 15,574 SNPs. The BLINK model was used because of the ease with which false discovery rate can be controlled in this model (Huang et al., 2018). The information about population structure obtained from STRUCTURE (*K* = 2) was used as a covariate. In this model, kinship was derived from pseudo‐QTN information. Bonferroni correction was applied to the calculated *p*‐values. The significance thresholds were, thus, set at 0.05/*n* and 0.01/*n* where “*n*” is the number of markers used. The GEA analysis was performed on the 281 genotypes with 88,804 SNPs after the recalculation of minor allele frequency in GAPIT.

The genes attributed to each marker by snpEff were used to identify the related protein families with the UniProtKB (The UniProt Consortium, 2021) database. If several results were available, only one was retained. If the corresponding protein was unknown, we used the molecular function attributed by IEA:InterPro (Camon et al., 2005) or the manual assertion based on the work of Gaudet et al. (2011).

## 3 RESULTS

The long‐read sequencing of *V*. *berlandieri* resulted in 1,620,094 reads >1 kb and a coverage of 50×. The N50 of reads was 15.6 kb and L50 was 680,049, indicating that 680,049 reads in which the smallest size was 15.6 kb were enough to obtain half of the 25,000,009,766 bases sequenced. In addition, 91% of reads had a length between 10 and 20 kb. Then, 2789 contigs were obtained with a N50 of 22.26 Mb and L50 of 9. When comparing with the *V*. *riparia* genome (Girollet et al., 2019), the *V*. *berlandieri* genome was well represented with 29 contigs. In the final version, 10 chromosomes presented a single contig, eight chromosomes had two contigs, and one chromosome was divided into three contigs. In total, 39,250 of the 42,413 genes from the Pinot noir reference genome (92.5%) were found in *V*. *berlandieri*. This high‐quality assembly resulted in a highly reliable reference genome for these analyses.

### 3.1 Population structure

**FIGURE 1:**
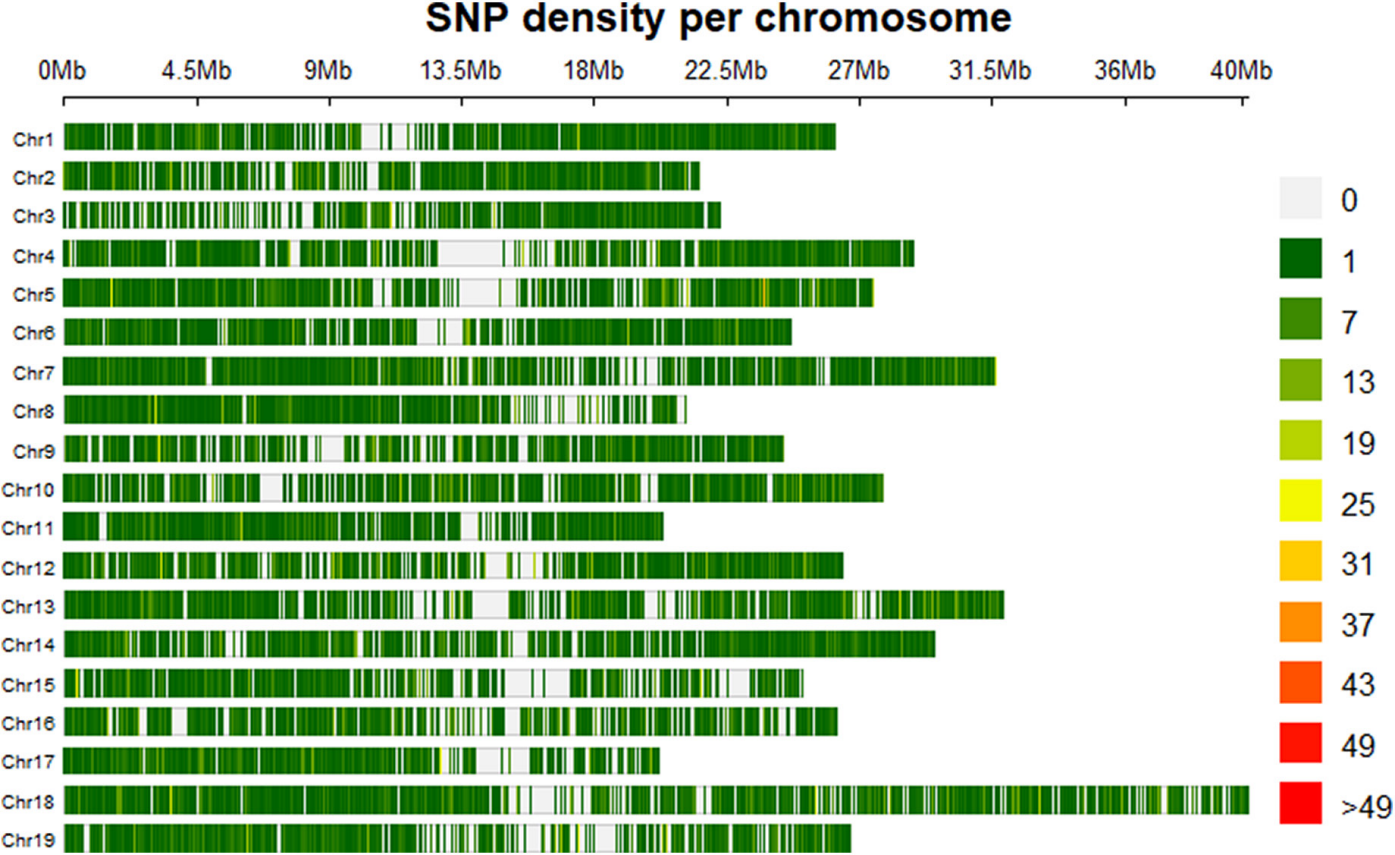
SNP density per kb obtained by GBS for each chromosome of the *Vitis berlandieri* genome.

**FIGURE 2:**
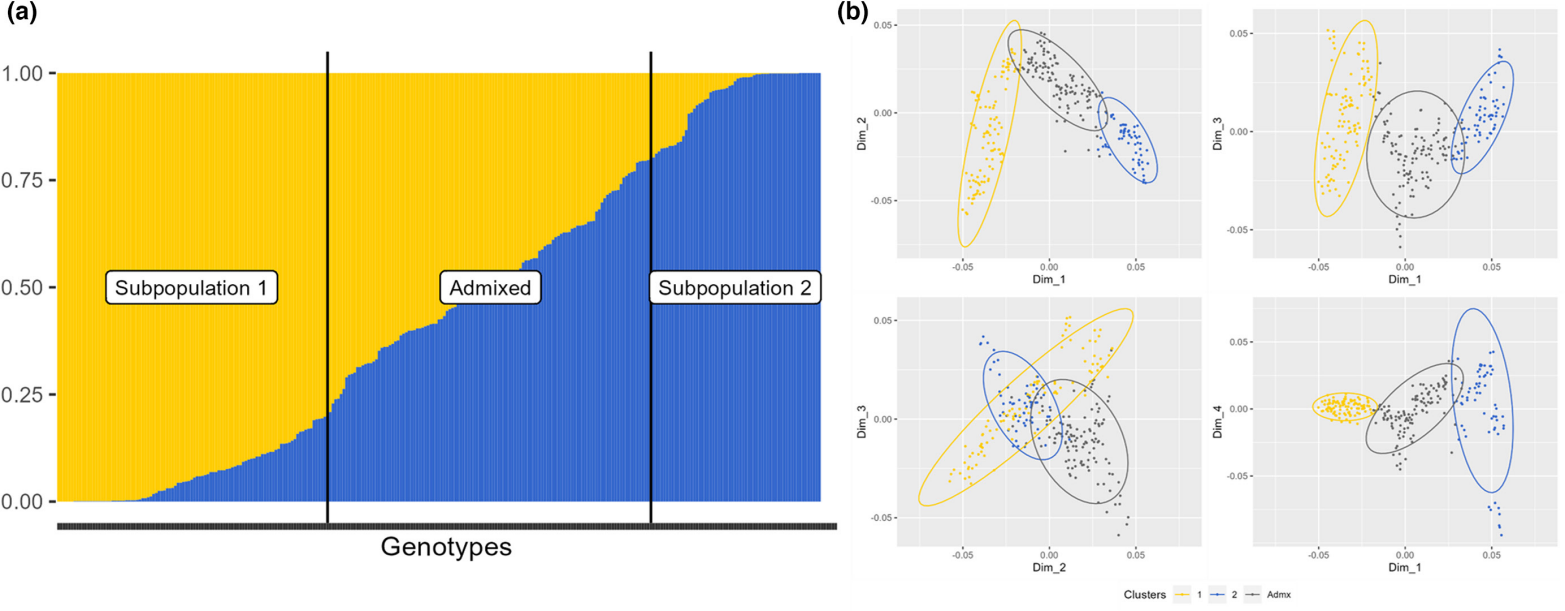
Population structure results from (a) STRUCTURE *K* = 2. The proportion of each genotype found in the two populations is shown. Whenever a genotype has a probability of belonging to a population of 80% or more, the genotype is attributed to that population, each subpopulation being separated by a vertical black line. (b) PCA based on SNP information, the first four principal components are represented and all genotypes are plotted and colored according to STRUCTURE groups; PC 1–4 explained 4%, 2%, 2%, and 1% of the variability, respectively.

The genotyping by sequencing of the *V*. *berlandieri* population allowed us to get 104,378 relevant SNPs with an average of 5500 SNPs per chromosome (Figure 1) and a mean of 0.2 SNPs per kb. From the marker set, 787 SNPs were detected with a high impact by snpEff, 17,388 SNPs with a low effect, and 18,215 with a moderate effect (Table S1). According to the results obtained with snpEff, most of SNPs were upstream_gene_variant (23,002), missense_variant (18,102), synonymous_variant (15,937), downstream_gene_variant (15,028), intron_variant (14,022), or intergenic_variant (10,622; Table S2). The optimal number of subpopulations determined by STRUCTURE was *K* = 2 according to the method of Evanno et al. (2005) (burn‐in = 20,000 and no. of MCMC iterations = 20,000). The two subpopulations were explored in greater depth (burn‐in = 100,000 and no. of MCMC iterations = 100,000; Figure 2a); subpopulation 1 was found to contain 100 genotypes, subpopulation 2 contained 63 genotypes, and the admixed subpopulation contained 118 genotypes.

The *k*‐means clustering method suggested that there were more subdivisions within the population, resulting in at least five subpopulations, as suggested by the sum of squares curve (Figure S1). The subpopulations were also explored by PCA, with the genotypes colored according to the groups previously determined with STRUCTURE, as described by Evanno et al. (2005) with the admixed group considered as a subpopulation, *K* = 3, (Figure 2b) and according to the results obtained with the *k‐*means clustering method (Hartigan & Wong, 1979; *K* = 5, Figure S2). The PCA (Figure 2b) revealed a clear subdivision into groups for *K* = 3, but the proportions of the variance explained by principal components 1, 2, and 3 were very low, at 3%, 2%, and 1%, respectively. For *K* = 5 (Figure S2), a clear separation of the groups was observed for dimensions 1/2 and 1/3. However, when dimensions 2/3 or 1/4 were used, group 3 was the only group that could be distinguished clearly. The IBD analysis revealed a correlation between genetic distance and physical distance between sampling points (Spearman's rho = 0.33 and *p*‐value = 10^−04^).

### 3.2 Linkage disequilibrium and genetic load

The extent of LD decay (*r* ^2^ < 0.2) was calculated per chromosome; it ranged from 307 bp on chromosome 16 (Figure S3a) to 8 kb on chromosome 6 (Figure S3b) with a mean value of 2.2 kb (method from Hill & Weir, 1988). SNPs with a predicted *high* impact accounted for 0.75% of SNPs, and these SNPs were the least represented group. Similar proportions of SNPs were predicted to have *moderate* and *low* impacts (ca. 17%; Table S1). SNPs classified as *modifiers* were the most abundant (65%). The distribution of each category of SNPs was similar between chromosomes. The number of SNPs for each estimated effect is detailed in Table S2. There were no significant differences in the accumulation of deleterious alleles between subpopulations (Figure S4).

### 3.3 Genome–environment association

**FIGURE 3:**
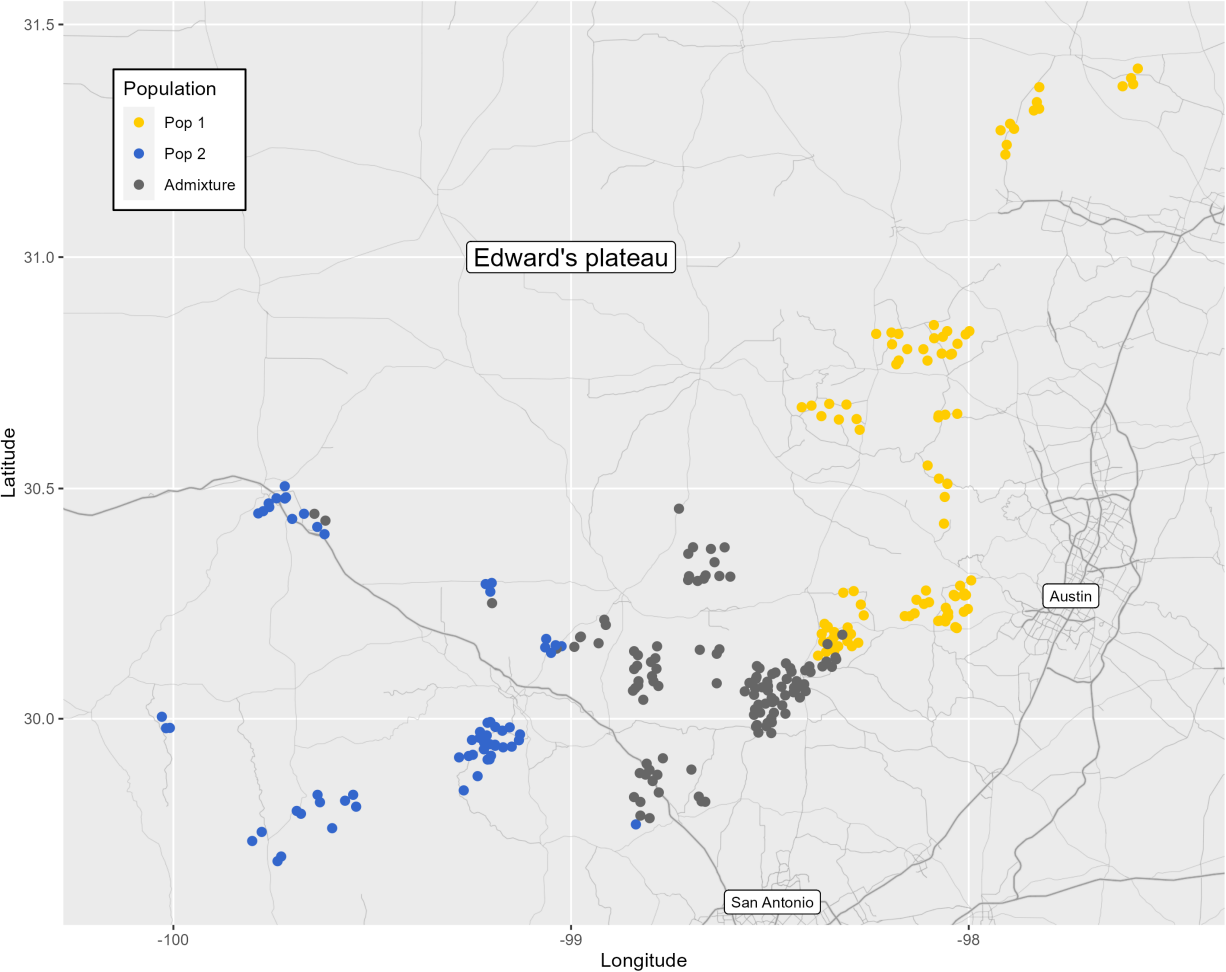
Geographic position of the sites from which plants were sampled in Texas (the genotypes from the same mother plant have been jittered to facilitate observation).

**FIGURE 4:**
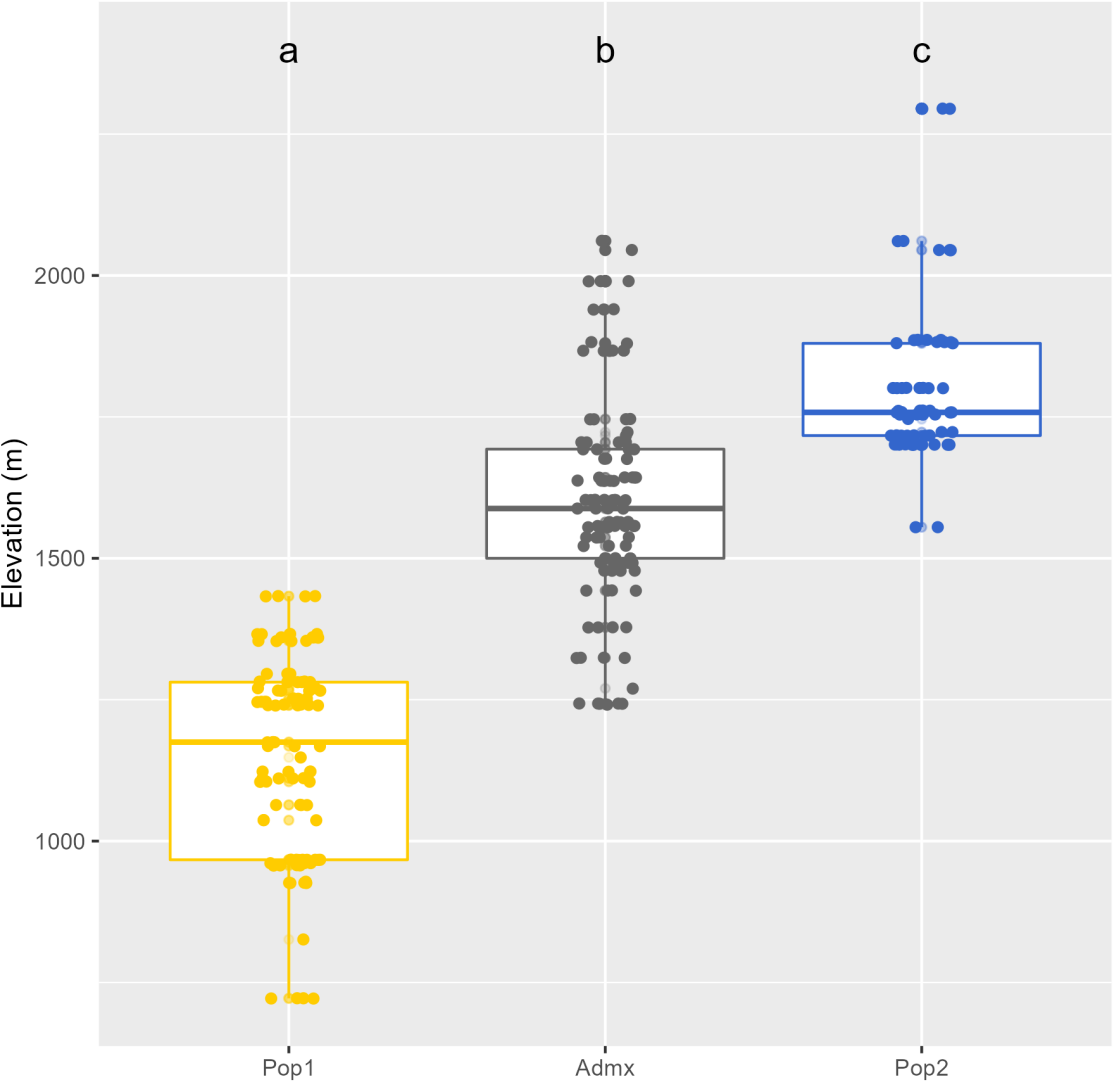
Boxplot of the elevations of the various subpopulations identified by STRUCTURE.

**FIGURE 5:**
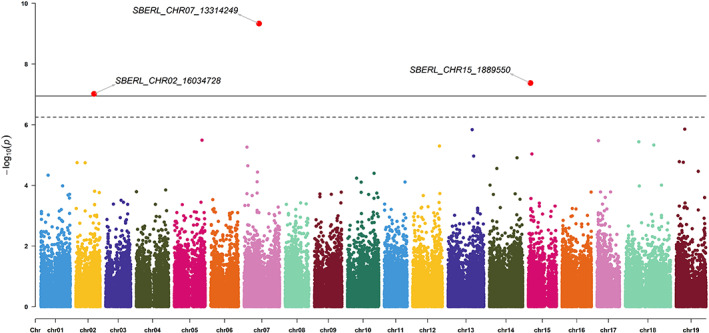
Manhattan plot for SNP associations with elevation. The thresholds were calculated by the Bonferroni method (*α*/NSNP) for *α* = 0.05 (dotted line) and 0.01 (solid line). Significant signals are highlighted in red, and the corresponding QQ plot is presented in Figure S9.

The structure of the population followed a geographic pattern (Figure 3). The elevation of the sampling sites differed considerably (Figure S5) ranging from 722 to 2295 m. The mean elevation of each subpopulation made it possible to separate the groups easily (Figure 4). Three QTL for elevation were identified on chromosomes 2 (*p*‐value = 9.60 × 10^−08^), 7 (*p*‐value = 4.24 × 10^−08^), and 15 (*p*‐value = 4.61 × 10^−10^; Figure 5). Considerable variation was observed for environmental variables, with GSR ranging from 395 to 581 mm, HYB from 6932 to 9326 and DI ranging from −139 to −29 (Figure S6).

Subpopulation 1 was located in an area that was warmer and damper than the areas occupied by the other two subpopulations, with a higher GST49, GSR49, RRSPR, HST, WFR, and SFR. The HI value revealed a *very warm climate* (>3000) for all subpopulations and was significantly higher for subpopulation 1 than for the other populations (Figure S6j); the CI value (>18) indicated *warm nighttime* conditions for subpopulation 1.

Subpopulation 2 was located in a region that was drier and cooler than that occupied by subpopulation 1, with a moderate risk of frost during the spring (<12°C; Figure S6h) and temperate nights (14–18°C; Figure S6k). Based on DI, the three subpopulations were considered to come from a *moderately dry* area, with a few genotypes from subpopulation 2 and the admixed subpopulation classified as coming from a *very dry* area, significantly drier than that for subpopulation 2 as a whole (Figure S6l).

**FIGURE 6:**
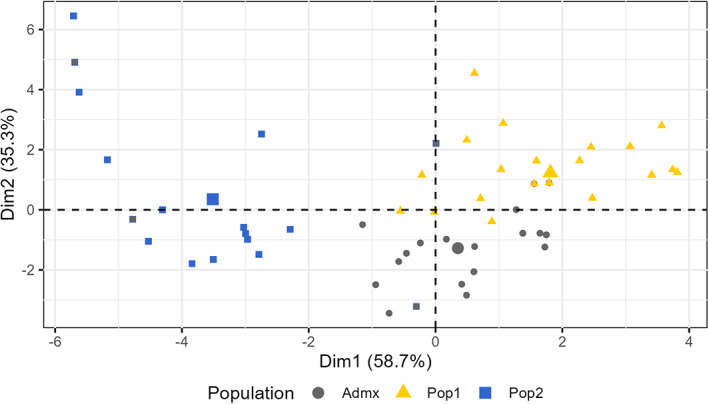
PCA of the environmental parameters accessed via the TerraClimate platform (Abatzoglou et al., 2018). The following variables were extracted: growing season temperature between April and September (GST49, GST, Jones, 2006), growing season rainfall between April and September (GSR49, GSR, Bois et al., 2017), springtime rainfall (RRSPR, from April to July), Branas hydrothermal index (HYB, Branas et al., 1946), winter cold damage index (WFR, Bois et al., 2014) spring frost index (SFR, Bois et al., 2014), heat stress index (HST, Bois et al., 2014), Huglin index (HI, Tonietto & Carbonneau, 2004), cool night index (CI, Tonietto & Carbonneau, 2004), and dryness index (DI, Tonietto & Carbonneau, 2004). The PC1 is mainly explained by SFR and WFR. The PC2 is mainly explained by HSI, HI, and DI. Each group identified by STRUCTURE is indicated.

**FIGURE 7:**
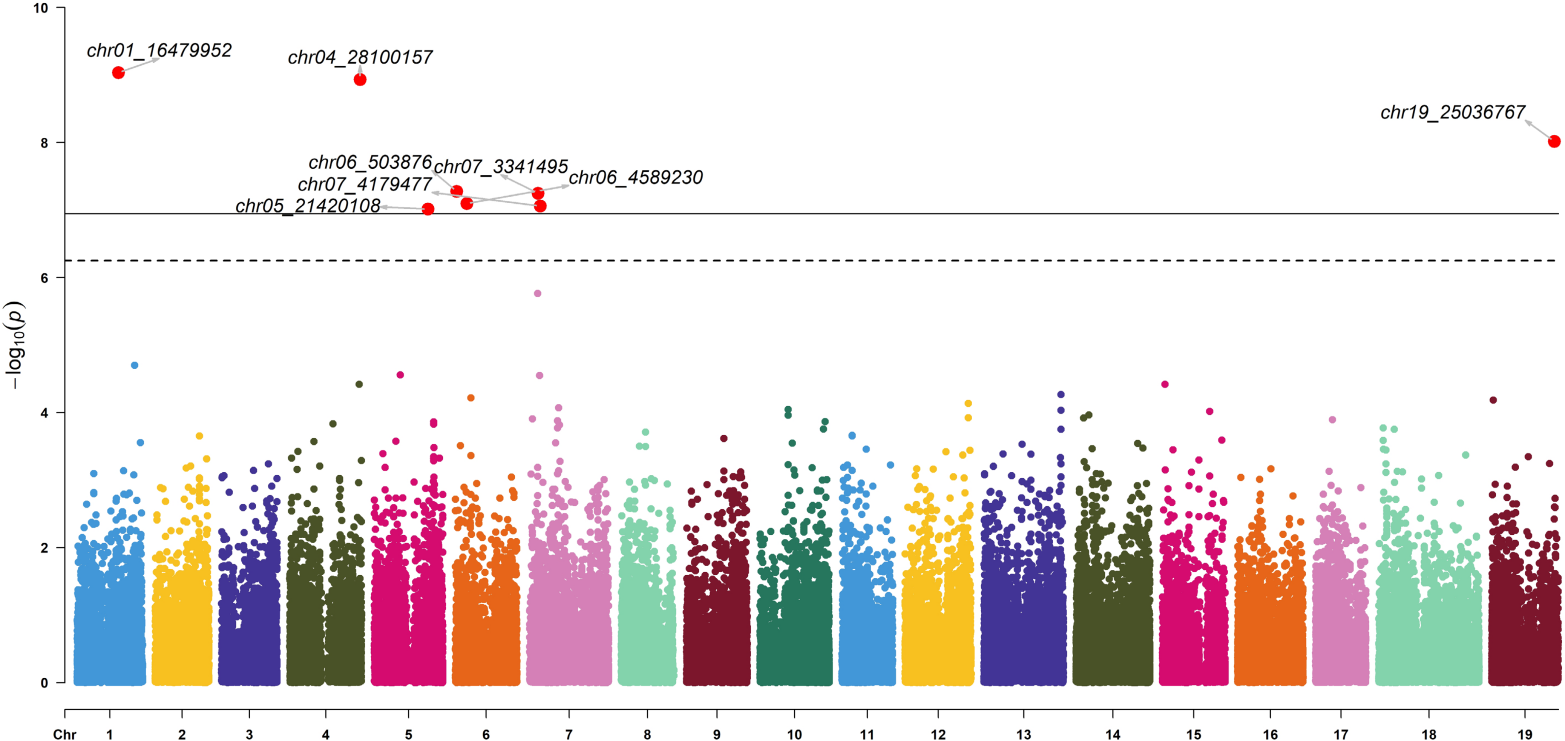
Manhattan plot for the PC_1 trait (principal component from the PCA on environmental parameters). The thresholds were calculated by the Bonferroni method (*α*/NSNP) for *α* = 0.05 (dotted line) and 0.01 (solid line). Significant signals are highlighted in red. The corresponding QQ plot is presented in Figure S10a.

**FIGURE 8:**
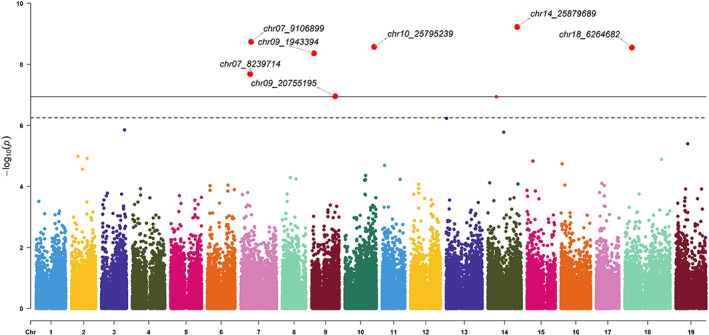
Manhattan plot for the PC_2 trait (principal component from the PCA on environmental parameters). The thresholds were calculated by the Bonferroni method (*α*/NSNP) for *α* = 0.05 (dotted line) and 0.01 (solid line). Significant signals are highlighted in red. The corresponding QQ plot is presented in Figure S10b.

As correlations were found between environmental parameters, PCA was performed to capture environmental variability in an integrated manner (Figure 6). The first two principal components (PC) explained 94% of the variance. The PC1 was principally explained by the parameters SFR and WFR which were related to frost risk during winter and spring. The PC2 was explained by HSI, HI, and DI which were related to high temperature and water availability. An analysis of the genome–environment association for the PC1 trait identified eight significant QTL on chromosomes 1, 4, 5, 6, 7 (2), and 19 (Figure 7). For PC2, seven significant QTL were detected on chromosomes 7 (2), 9 (2), 10, 14, and 18 (Figure 8). The significant QTL corresponded to SNPs classified as *modifiers*, or with *low* or *moderate* impact, and the genes concerned had basic molecular functions potentially involved in various biological pathways (Table 1). For each marker, the effect of each allele in the homozygous and heterozygous states have been explored (Figure S12).

**TABLE 1:** Markers associated with environmental parameters.

Trait	Chr	Position	*n*	*p*‐Values	maf	Effect	Impact	Gene	Molecular function
Alt	chr02	16034728	281	9.60E‐08	0.25	5_prime_UTR_variant	MODIFIER	Vitvi02g00558	Asparagine‐tRNA ligase
Alt	chr07	13314249	281	4.61E‐10	0.07	missense_variant	MODERATE	Vitvi07g02904	Multiple‐splicing variant
Alt	chr15	1889550	281	4.24E‐08	0.33	downstream_gene_variant	MODIFIER	Vitvi15g01070	Ferric reduction oxidase 2
PC1	chr01	16479952	277	9.20E‐10	0.06	3_prime_UTR_variant	MODIFIER	Vitvi01g00842	Unknown protein
PC1	chr04	28100157	277	1.17E‐09	0.08	intron_variant	MODIFIER	Vitvi04g01724	Multiple‐splicing variant
PC1	chr05	21420108	277	9.64E‐08	0.08	3_prime_UTR_variant	MODIFIER	Vitvi05g02116	Glycosyltransferase
PC1	chr06	503876	277	5.29E‐08	0.31	missense_variant	MODERATE	Vitvi06g01533	Nitrilase
PC1	chr06	4589230	277	8.01E‐08	0.47	upstream_gene_variant	MODIFIER	Vitvi06g01246	RNA binding
PC1	chr07	3341495	277	5.63E‐08	0.09	downstream_gene_variant	MODIFIER	Vitvi07g01826	Metal ion binding
PC1	chr07	4179477	277	8.71E‐08	0.10	downstream_gene_variant	MODIFIER	Vitvi07g01729	(BHLH domain‐containing protein) Protein dimerization activity
PC1	chr19	25036767	277	9.65E‐09	0.06	intron_variant	MODIFIER	Vitvi19g01666	Chromatin binding
PC2	chr07	8239714	277	2.07E‐08	0.08	5_prime_UTR_variant	MODIFIER	Vitvi07g01381	Pyrimidine nucleotide‐sugar Transmembrane transporter activity
PC2	chr07	9106899	277	1.81E‐09	0.12	synonymous_variant	LOW	Vitvi07g02508	Nuclear localization sequence binding
PC2	chr09	1943394	277	4.32E‐09	0.42	upstream_gene_variant	MODIFIER	Vitvi09g01386	Multiple‐splicing variant
PC2	chr09	20755195	277	1.10E‐07	0.07	missense_variant	MODERATE	Vitvi09g01590	PGG domain‐containing protein
PC2	chr10	25795239	277	2.68E‐09	0.19	missense_variant	MODERATE	Vitvi10g00114	Transmembrane transporter activity
PC2	chr14	25879689	277	5.90E‐10	0.06	splice_region_variant&intron_variant	LOW	Vitvi14g01620	Glycosyltransferase activity
PC2	chr18	6264682	277	2.80E‐09	0.09	downstream_gene_variant	MODIFIER	Vitvi18g00553	Transcription factor

## 4 DISCUSSION

De novo whole‐genome sequencing of *V*. *berlandieri* made it possible to explore the genetic structure of a wild *V*. *berlandieri* population with genome‐wide molecular markers. We identified two distinct subgroups that could be explained by isolation by distance. However, no significant differences in genetic diversity or genetic load were found between subpopulations. Using geographic coordinates and climatic data from meteorological stations, we characterized the environments from which the subpopulations were collected and identified several QTL associated with environmental conditions and agronomic indices of climatic conditions.

The complexity of the plant genome renders short‐read sequencing highly challenging, due to the large numbers of repetitive sequences and heterozygosity (Schatz et al., 2012). We used long‐read sequencing, which overcomes these problems, in this study. The low level of LD in wild *Vitis* species is well known (Liang et al., 2019; Marrano et al., 2018; Myles et al., 2010, 2011; Péros et al., 2021). Our results confirm the rapid LD decay (about 2 kb) reported in previous studies. This rapid LD decay drives the retention of as many SNPs as possible, making it possible to capture a maximum of the genetic variability in the population (Flutre et al., 2019; Myles et al., 2011; Remington et al., 2001). We obtained a set of 104,378 SNPs, which we used for fine genetic association studies. We ensured that the maximum level of genetic diversity was retained, by including genotypes with up to 60% missing data in our analyses.

### 4.1 Genetic structure

Wild *Vitis* species are characterized by considerable genetic diversity (This et al., 2006). The genetic diversity and the relationships between these species and varieties has already been investigated, as interspecific hybrids and rootstocks have been shown to display greater genetic diversity than varieties of *V*. *vinifera* (de Andrés et al., 2007; Laucou et al., 2011). The wild grapevines (*V*. *vinifera* ssp. sylvestris) of the Anatolia region, which is considered to be the center of origin of grapevine (McGovern, 2003), are highly diverse (Ekhvaia et al., 2014; Ergül et al., 2011). However, the genetic diversity of wild grapevines in the European region is lower (Di Vecchi‐Staraz et al., 2009; Lopes et al., 2009), probably mirroring the human footprint on these populations. The genetic diversity of *Vitis* has been also explored in the Americas, revealing different groups in South America (Martinez et al., 2006) and North America (Péros et al., 2021), and considerable diversity between species. However, genetic diversity and genetic structure within *Vitis* species has been little explored. We addressed this issue here, by studying the population structure and genetic diversity in *V*. *berlandieri*, a species of considerable interest for the breeding of grapevine rootstocks.

The population structure detected in our study could be explained geographically. The two subpopulations were from physically close locations and were poorly differentiated (*F*_ST_ = 0.036 and 0.060 for subpopulations 1 and 2, respectively, in STRUCTURE analysis), probably due to the small size of the geographic area used for sampling. The significant IBD could explain the low *F*_ST_ observed in each subpopulation, as it could indicate existing gene flow between subpopulations. Moreover, the *F*_ST_ between the two subpopulations was 0.032. A low level of differentiation at regional level has also been observed in other perennial species, such as *Fagus sylvatica* (Buiteveld et al., 2007; Pluess et al., 2016), *Prunus sibirica* (Wang et al., 2014), and *Malus sieversii* (Richards et al., 2009).

This poor differentiation between subpopulations reveals the minor impact of this genetic structure, reducing the risk of false‐positive detection in GWAS models (Santure & Garant, 2018). The sampling area in our study was restricted to the region in which *V*. *berlandieri* is endemic, but this species extends over a larger area (southern New Mexico, south‐western Texas and northern Mexico; Galet, 1988) with different soil and climatic conditions. Greater diversity might, therefore, be expected for a larger sampling area. The two non‐admixed subpopulations could be characterized as a “northern” and a “southern” subpopulation, with a significantly different mean elevation between these two subpopulations (Figure 4).

The five clusters were well distinguished geographically (Figure S7), but elevation divided the total population into three, rather than five subpopulations (Figure S8). We, therefore, considered a genetic structure based on two subpopulations for this geographical area (plus an admixed subpopulation). We found a significant correlation between genetic relatedness and physical distance that was explained by a phenomenon of isolation by distance, consistent with the difference in elevation between the two subpopulations. We took the significant results for IBD into account in the GEA analysis, using kinship information to control for false discovery rate. IBD may also have affected the STRUCTURE results, leading to the detection of false genetic subgroups (Perez et al., 2018). In this study, genetic structure was also controlled by incorporating the proportions of ancestry for each genotype from STRUCTURE.

According to Kimura's neutral theory of evolution (1968), most new mutations in a population are deleterious or neutral. The number of deleterious alleles may be higher in domesticated plants and animals than in wild species (Wallace et al., 2018). Hill–Robertson interference may result in an accumulation of deleterious mutations in genomes, reducing the efficacy of selection (Hill & Robertson, 1966). Deleterious variants are hard to predict in plants (Kono et al., 2018). We used snpEff, to predict deleterious variants and to investigate their relationship to genetic structure. We found no difference in the proportion of deleterious alleles between the *V*. *berlandieri* subpopulations. We were therefore unable to confirm that the admixed subpopulation had a lower genetic load due to greater mixing (Peischl et al., 2013). However, other sequence‐based estimates, such as the efficacy of selection (Chen et al., 2017), may shed light on the ways in which natural selection deals with the accumulation of deleterious mutations. The link between domestication and the accumulation of deleterious mutations remains unclear, but this knowledge might be directly useful in breeding programs. Günther and Schmid (2010) classified 20% of polymorphic sites as deleterious variants in rice and *Arabidopsis* and found the genetic load to be lower in wild rice than in domesticated rice, consistent with the hypothesis of a “cost of domestication” (Lu et al., 2006). However, M.S. Kim et al. (2021) reported that the deleterious variant burden was lower in domesticated soybean than in wild soybean. Domestication has different impacts on annual and perennial plants (Gaut et al., 2015), because perennials are mostly propagated vegetatively, limiting the “cost of domestication”. Nevertheless, vegetative propagation has resulted in the fixation of deleterious mutations in cassava (Ramu et al., 2017) and grapevine (Zhou et al., 2017), both of which are both perennials.

### 4.2 Adaptation to the environment

The sampling area in Texas is known locally as “The Hill Country,” highlighting the alternation between valley and hills. Its vegetation changes with elevation and the various environmental pressures would also be expected to vary with elevation, accounting for the variation of a large set of environmental variables (e.g., temperature, humidity, and edaphic conditions). For this reason, despite the smaller size of the area sampled in this study than in previous studies on grapevine (Aradhya et al., 2003; Bacilieri et al., 2013; Péros et al., 2011, 2015, 2021), we were able to highlight the existence of different climatic regions for the three subpopulations. Subpopulation 1 occurred in a hot and humid area, probably resulting in higher biotic pressure (Bois et al., 2014), with more efficient photosynthesis and secondary metabolite synthesis (Tonietto & Carbonneau, 2004). Subpopulation 2 was found in an area with a cooler climate and lower rainfall levels during the growing period, resulting in a larger water deficit. Different environmental pressures would be expected to apply in these areas of different climatic conditions, potentially leading to insular subpopulation adaptation. This result is consistent with the previous observations of Rives (1974), who reported strong phenotypic diversity between wild species, such as *V*. *riparia*, *V*. *rupestris*, and *V*. *berlandieri*, growing in Texas, USA, in terms of morphology, pathogen tolerance, and precocity. In particular, Rives reported that *V*. *berlandieri* plants from the north had hairy leaves and ribbed shoots, whereas those from the south were almost glabrous, with smooth stems. These observations are consistent with our results revealing two gene pools in this species. In our study, the climatic parameter extracted from the Terraclimate platform followed the same directional gradient, thus, the coordinate of each genotype along the two axis of a PCA were used as indicators of the overall environmental variability. The principal components coordinates were used in a GEA and considered the environmental conditions experienced by the genotypes. The detected markers associated with environmental variability may reflect genetic regions relating to local adaptation (Williams, 1996) with potential for use as indicators for the prediction of phenotypic variation for adaptive traits (Lasky et al., 2015). The markers associated with PC1 were mostly related to frost risk during winter and spring (SFR and WFR), whereas the markers associated with PC2 were mostly associated with heat stress and water availability (HSI, HI, and DI). GEA studies have detected associations between genetic factors and specific environmental conditions in nature, revealing markers responsible for driving local adaptation or “ecoclines” (Huxley, 1938). This method led to the detection of QTL associated with broad environmental traits in *Arabidopsis* (Frachon et al., 2018), barley (Chang et al., 2022), sorghum (Lasky et al., 2015; Menamo et al., 2021), sunflower (Todesco et al., 2020), bean (Ariani & Gepts, 2019; Elias et al., 2021), five alpine Brassicaceae species (Zulliger et al., 2013), and strawberry (Hu et al., 2022). Moreover, these markers can be genotyped in germplasm collections to improve estimates of the genetic diversity present in the collection and to select the best candidates for breeding programs. In this study, 18 SNPs associated with environmental traits were highlighted. The GEA analysis identified three QTL associated with sampling site elevation. The sequence of chr02_16034728 is related to an asparagine‐tRNA ligase (Schimmel, 1987). Asparagine is involved in nitrogen transport in plants and asparagine accumulation is induced by multiple stresses, including mineral deficiencies, drought, salt, toxic metals, and pathogen attack (Lea et al., 2007). The chr07_13314249 marker is associated with multiple‐splicing variants and chr15_1889550 matches with AT1G01580 in the *A*. *thaliana* genome, which has been shown to be related to ferric reduction oxidase 2 (Kim et al., 2019). Iron is involved in chlorophyll biosynthesis, photosynthesis, and nucleotide synthesis (Kim & Guerinot, 2007). Elevation would be expected to affect a large set of environmental variations linked to a number of different metabolic pathways. The PCA on environmental parameters extracted from the TerraClimate platform revealed associations with general environmental variability in each subpopulation. Eight QTL were identified for PC1, including chr05_21420108, which is linked to glycosyltransferase activity (Ramasamy et al., 2005) and mostly affects sugar metabolism (Keegstra & Raikhel, 2001) and chr06_503876, which is related to nitrilase enzyme synthesis (Piotrowski & Volmer, 2006). Nitrilases have been reported to be involved in plant–microbe interactions (Howden & Preston, 2009). chr06_4589230 is involved in RNA binding, chr07_3341495 is thought to be involved in metal ion binding and chr19_25036767 is thought to be involved in chromatin binding (Gaudet et al., 2011). Seven QTL involved in diverse metabolic activities have been detected for PC2: pyrimidine nucleotide‐sugar transmembrane transporter activity (chr07_8239714, Hadley et al., 2014), nuclear localization sequence binding (chr07_9106899, Gaudet et al., 2011), transmembrane transporter activity (chr10_25795239, Camon et al., 2005), and a transcription factor (chr18_6264682, Gaudet et al., 2011). Like chr05_21420108 for PC1, chr14_25879689 is associated with glycosyltransferase activity (Camon et al., 2005). Finally, some of the metabolic pathways identified in our GEA study, such as sugar, iron, and asparagine metabolism, may be involved in adaptation.

The genotyping of *V*. *berlandieri* germplasm collections for the molecular markers identified here is of potential interest for the selection of individuals carrying favorable alleles for a particular environmental stressor, to facilitate the selection of genetic resources better adapted to local cultivation environments. Here, we identified 18 QTL as correlated with environment variability; two had high *F*_ST_ values—0.189 and 0.221 for chr04_28100157 (detected in GEA for PC1) and chr07_9106899 (detected in GEA for PC2), respectively—corresponding to strong genetic differentiation between subpopulations at these loci (Weir & Clark Cockerham, 1984; Wright, 1984). However, these results require confirmation by further studies based on genetic engineering or the testing of these genotypes in a common garden.

## 5 CONCLUSION

Our results confirm the existence of considerable genetic diversity in the *V*. *berlandieri* genetic background, even over a relatively small sampling area (about 40,000 km^2^). The analysis of genome–environment association revealed genetic markers associated with the climatic conditions in the sampling areas. These associations should highlight genetic regions involved in the adaptation of plants to different environmental conditions. Wild genotypes constitute valuable resources that could be subjected to more precise selection and included in breeding programs. However, before their inclusion in breeding programs, these genotypes will need to undergo assessments of their agronomic performance.

## CONFLICT OF INTEREST STATEMENT

The authors have no conflict of interest to declare.

## Supporting information


Appendix S1.

## Data Availability

DNA sequence of the *V*. *berlandieri* referene genome: Data openly available in a public repository that does not issue DOIs: NCBI, BioProject ID: PRJNA886625. DNA sequences of the genotyping by sequencing of the *V*. *berlandieri* population: Data openly available in a public repository that does not issue DOIs: NCBI, BioProject ID: PRJNA886619. Other data: Data available on request from the authors.
