# Supplementary material for: Genetic structure and first genome‐wide insights into the adaptation of a wild relative of grapevine, Vitis berlandieri
Source: Evol Appl. 2023 Jun 9;16(6):1184–200. doi: 10.1111/eva.13566 (PMC10286229; doi:10.1111/eva.13566)
Supplement: Supplementary file 1 — Appendix S1. [file EVA-16-1184-s001.docx]

**Supplemental data**

Suppl. Table 1: Impact of SNPs predicted by the snpEff^®^ program as a function of position in the genome: i) *high* if they cause a loss of protein function, ii) *moderate* if they alter protein effectiveness, iii) *low* if they have no impact on the protein and iv) *modifier* for non-coding variants. The number of SNPs for each impact category is indicated per chromosome.

| Impact | chr01 | chr02 | chr03 | chr04 | chr05 | chr06 | chr07 | chr08 | chr09 | chr10 | chr11 | chr12 | chr13 | chr14 | chr15 | chr16 | chr17 | chr18 | chr19 | Total |
| --- | --- | --- | --- | --- | --- | --- | --- | --- | --- | --- | --- | --- | --- | --- | --- | --- | --- | --- | --- | --- |
| HIGH | 30 | 25 | 30 | 36 | 67 | 32 | 62 | 20 | 39 | 45 | 34 | 42 | 59 | 70 | 40 | 47 | 24 | 47 | 38 | 787 |
| LOW | 903 | 684 | 815 | 957 | 1169 | 841 | 1233 | 747 | 710 | 836 | 699 | 959 | 1204 | 1247 | 678 | 874 | 736 | 1174 | 922 | 17388 |
| MODERATE | 853 | 683 | 857 | 841 | 1543 | 785 | 1203 | 728 | 685 | 937 | 679 | 913 | 1393 | 1406 | 806 | 954 | 653 | 1244 | 1052 | 18215 |
| MODIFIER | 3630 | 3029 | 2987 | 3347 | 4085 | 2900 | 4669 | 2967 | 3545 | 3835 | 2855 | 3961 | 4634 | 4416 | 2954 | 2885 | 2692 | 5011 | 3586 | 67988 |
| Total | 5416 | 4421 | 4689 | 5181 | 6864 | 4558 | 7167 | 4462 | 4979 | 5653 | 4267 | 5875 | 7290 | 7139 | 4478 | 4760 | 4105 | 7476 | 5598 | 104378 |

Suppl. Table 2: Effect of SNPs predicted by snpEff® software according to position in the genome. The number of SNPs belonging to each effect category is indicated by chromosome.

| **Effect** | **chr1** | **chr2** | **chr3** | **chr4** | **chr5** | **chr6** | **chr7** | **chr8** | **chr9** | **chr10** | **chr11** | **chr12** | **chr13** | **chr14** | **chr15** | **chr16** | **chr17** | **chr18** | **chr19** | **Total** |
| --- | --- | --- | --- | --- | --- | --- | --- | --- | --- | --- | --- | --- | --- | --- | --- | --- | --- | --- | --- | --- |
| 3_prime_UTR_variant | 178 | 108 | 190 | 228 | 232 | 161 | 213 | 159 | 194 | 167 | 162 | 166 | 249 | 241 | 163 | 84 | 178 | 205 | 165 | **3443** |
| 5_prime_UTR_premature_start_codon_gain_variant | 15 | 14 | 9 | 24 | 17 | 18 | 26 | 21 | 12 | 17 | 7 | 16 | 16 | 21 | 11 | 7 | 17 | 30 | 16 | **314** |
| 5_prime_UTR_variant | 112 | 79 | 64 | 100 | 81 | 88 | 126 | 117 | 80 | 78 | 52 | 114 | 115 | 110 | 60 | 68 | 94 | 155 | 82 | **1775** |
| downstream_gene_variant | 822 | 681 | 660 | 838 | 1134 | 642 | 1096 | 615 | 737 | 809 | 643 | 758 | 897 | 900 | 627 | 603 | 589 | 1106 | 871 | **15028** |
| initiator_codon_variant | 0 | 0 | 0 | 1 | 1 | 0 | 0 | 0 | 0 | 0 | 1 | 0 | 0 | 0 | 0 | 0 | 0 | 1 | 0 | **4** |
| intergenic_region | 526 | 567 | 433 | 340 | 469 | 407 | 544 | 237 | 740 | 693 | 392 | 847 | 848 | 694 | 561 | 579 | 293 | 809 | 643 | **10622** |
| intragenic_variant | 0 | 0 | 0 | 0 | 0 | 0 | 0 | 24 | 0 | 0 | 0 | 0 | 0 | 0 | 0 | 0 | 0 | 0 | 0 | **24** |
| intron_variant | 818 | 602 | 570 | 804 | 842 | 663 | 1031 | 611 | 687 | 701 | 753 | 797 | 990 | 864 | 574 | 564 | 650 | 849 | 652 | **14022** |
| missense_variant | 850 | 674 | 853 | 833 | 1530 | 780 | 1202 | 725 | 680 | 932 | 677 | 909 | 1384 | 1396 | 802 | 944 | 649 | 1238 | 1044 | **18102** |
| missense_variant&splice_region_variant | 3 | 9 | 4 | 8 | 13 | 5 | 1 | 3 | 5 | 5 | 2 | 4 | 9 | 10 | 4 | 10 | 4 | 6 | 8 | **113** |
| non_coding_transcript_exon_variant | 6 | 9 | 0 | 0 | 0 | 0 | 7 | 0 | 1 | 15 | 9 | 3 | 15 | 0 | 3 | 0 | 2 | 2 | 0 | **72** |
| splice_acceptor_variant&intron_variant | 3 | 1 | 3 | 8 | 10 | 0 | 2 | 1 | 4 | 1 | 0 | 5 | 4 | 2 | 4 | 0 | 1 | 5 | 0 | **54** |
| splice_donor_variant&intron_variant | 0 | 3 | 1 | 4 | 5 | 3 | 1 | 1 | 7 | 4 | 3 | 2 | 3 | 1 | 0 | 2 | 2 | 2 | 3 | **47** |
| splice_region_variant |  | 1 | 3 | 2 | 2 | 3 | 9 | 3 | 3 | 3 | 1 | 5 | 1 | 2 | 1 | 0 | 1 | 3 | 1 | **44** |
| splice_region_variant&intron_variant | 48 | 31 | 49 | 47 | 48 | 54 | 73 | 44 | 37 | 39 | 35 | 50 | 58 | 64 | 32 | 40 | 38 | 52 | 45 | **884** |
| splice_region_variant &  non_coding_transcript_exon_variant | 0 | 0 | 0 | 0 | 1 | 0 | 0 | 0 | 0 | 0 | 1 | 1 | 0 | 0 | 2 | 0 | 0 | 0 | 1 | **6** |
| splice_region_variant&stop_retained_variant | 1 | 3 | 2 | 0 | 0 | 2 | 0 | 0 | 1 | 0 | 1 | 0 | 0 | 1 | 0 | 0 | 0 | 1 | 0 | **12** |
| splice_region_variant&synonymous_variant | 5 | 6 | 3 | 6 | 10 | 8 | 8 | 1 | 4 | 4 | 4 | 5 | 9 | 3 | 3 | 6 | 8 | 12 | 9 | **114** |
| start_lost | 1 | 0 | 0 | 2 | 0 | 1 | 4 | 1 | 2 | 0 | 0 | 1 | 0 | 1 | 2 | 2 | 0 | 0 | 0 | **17** |
| start_lost&splice_region_variant | 0 | 0 | 0 | 0 | 0 | 0 | 0 | 0 | 0 | 0 | 1 | 0 | 0 | 0 | 0 | 0 | 1 | 0 | 0 | **2** |
| stop_gained | 19 | 16 | 18 | 11 | 30 | 18 | 39 | 12 | 14 | 25 | 17 | 16 | 34 | 46 | 20 | 26 | 15 | 24 | 27 | **427** |
| stop_gained&splice_region_variant | 1 | 0 | 0 | 1 | 0 | 0 | 1 | 0 | 0 | 0 | 1 | 0 | 0 | 1 | 0 | 1 | 0 | 0 | 0 | **6** |
| stop_lost | 6 | 5 | 7 | 9 | 21 | 10 | 15 | 5 | 12 | 15 | 12 | 17 | 17 | 19 | 14 | 15 | 5 | 16 | 8 | **228** |
| stop_lost&splice_region_variant | 0 | 0 | 1 | 1 | 1 | 0 | 0 | 0 | 0 | 0 | 0 | 1 | 1 | 0 | 0 | 1 | 0 | 0 | 0 | **6** |
| stop_retained_variant | 2 | 2 | 1 | 0 | 8 | 4 | 4 | 3 | 4 | 2 | 3 | 5 | 1 | 10 | 5 | 4 | 3 | 8 | 4 | **73** |
| synonymous_variant | 832 | 627 | 748 | 877 | 1082 | 752 | 1113 | 675 | 649 | 771 | 646 | 877 | 1119 | 1146 | 624 | 817 | 669 | 1067 | 846 | **15937** |
| upstream_gene_variant | 1168 | 983 | 1070 | 1037 | 1327 | 939 | 1652 | 1204 | 1106 | 1372 | 844 | 1276 | 1520 | 1607 | 966 | 987 | 886 | 1885 | 1173 | **23002** |
| **Total** | **5416** | **4421** | **4689** | **5181** | **6864** | **4558** | **7167** | **4462** | **4979** | **5653** | **4267** | **5875** | **7290** | **7139** | **4478** | **4760** | **4105** | **7476** | **5598** | **104378** |


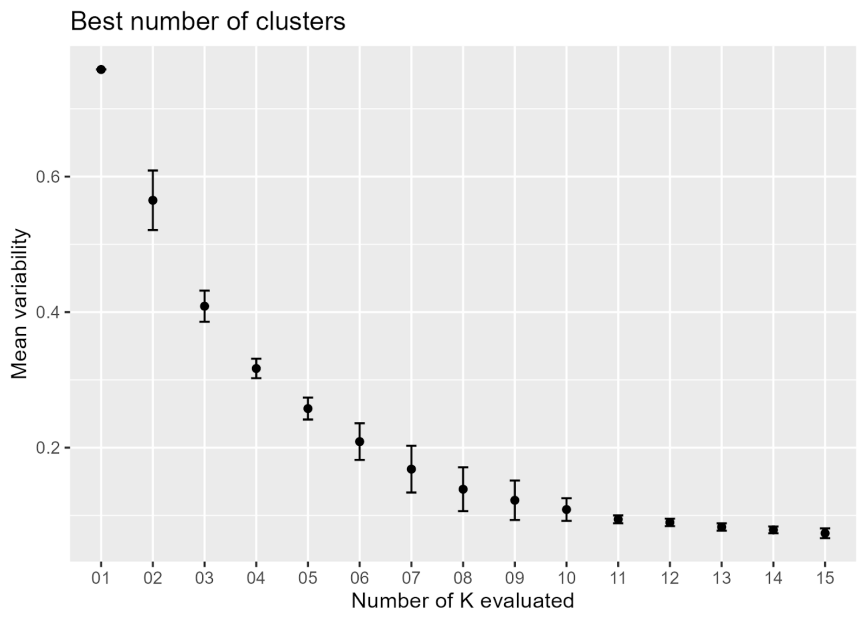


Suppl. Fig. 1: Mean variability of the within-cluster sum of squares according to the number of populations. The results were obtained by *k*-means clustering with the Hartigan and Wong (1979) algorithm


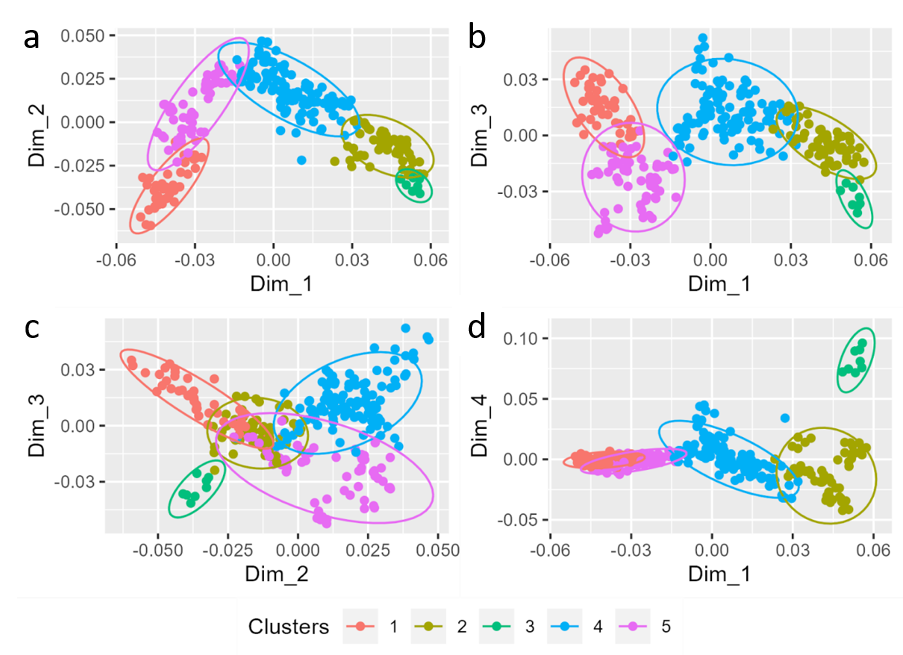


Suppl. Fig. 2: PCA of each genotype obtained with the 104378 SNPs. Groups are identified according to *k*-means clustering for K=5.


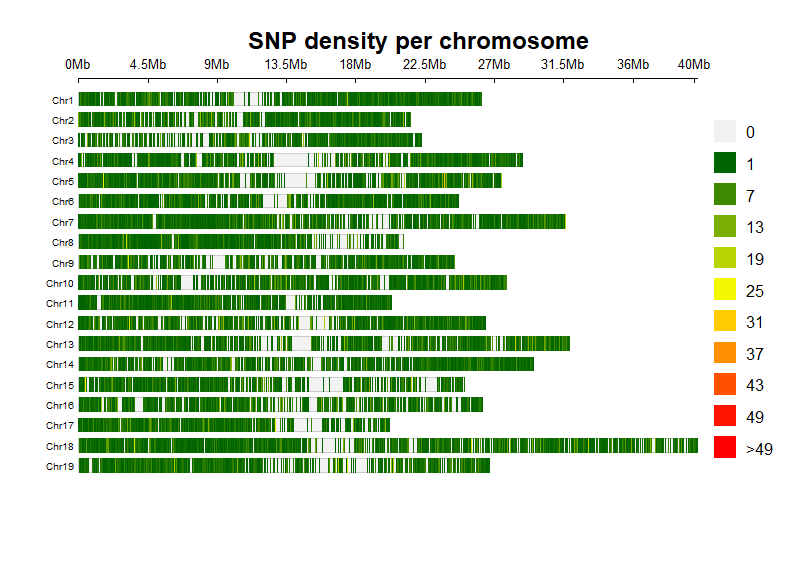


Suppl. Fig. 3: SNP density per kb obtained by GBS for each chromosome of the *V. berlandieri* genome.


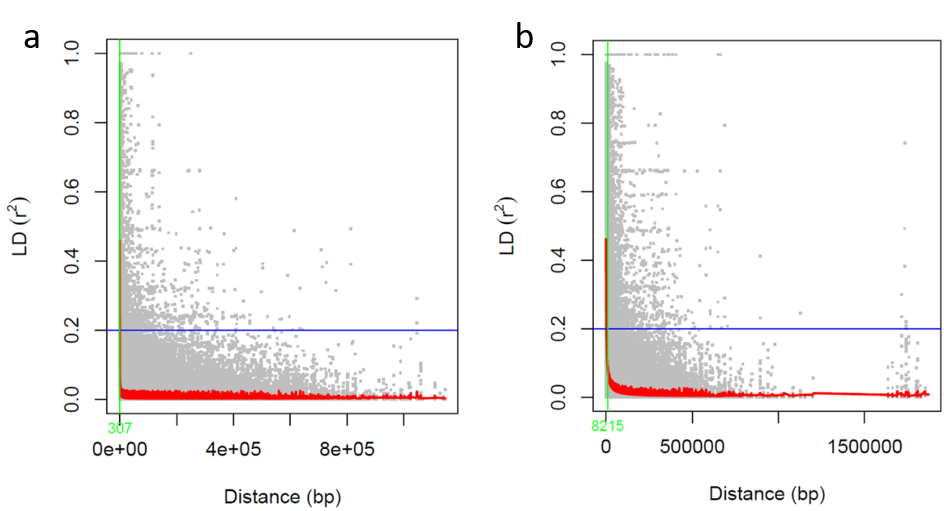


Suppl. Fig. 4: Linkage disequilibrium on chromosomes 16 (a) and 6 (b). Linkage disequilibrium is calculated for r²<0.2 as described by Hill and Weir (1988).


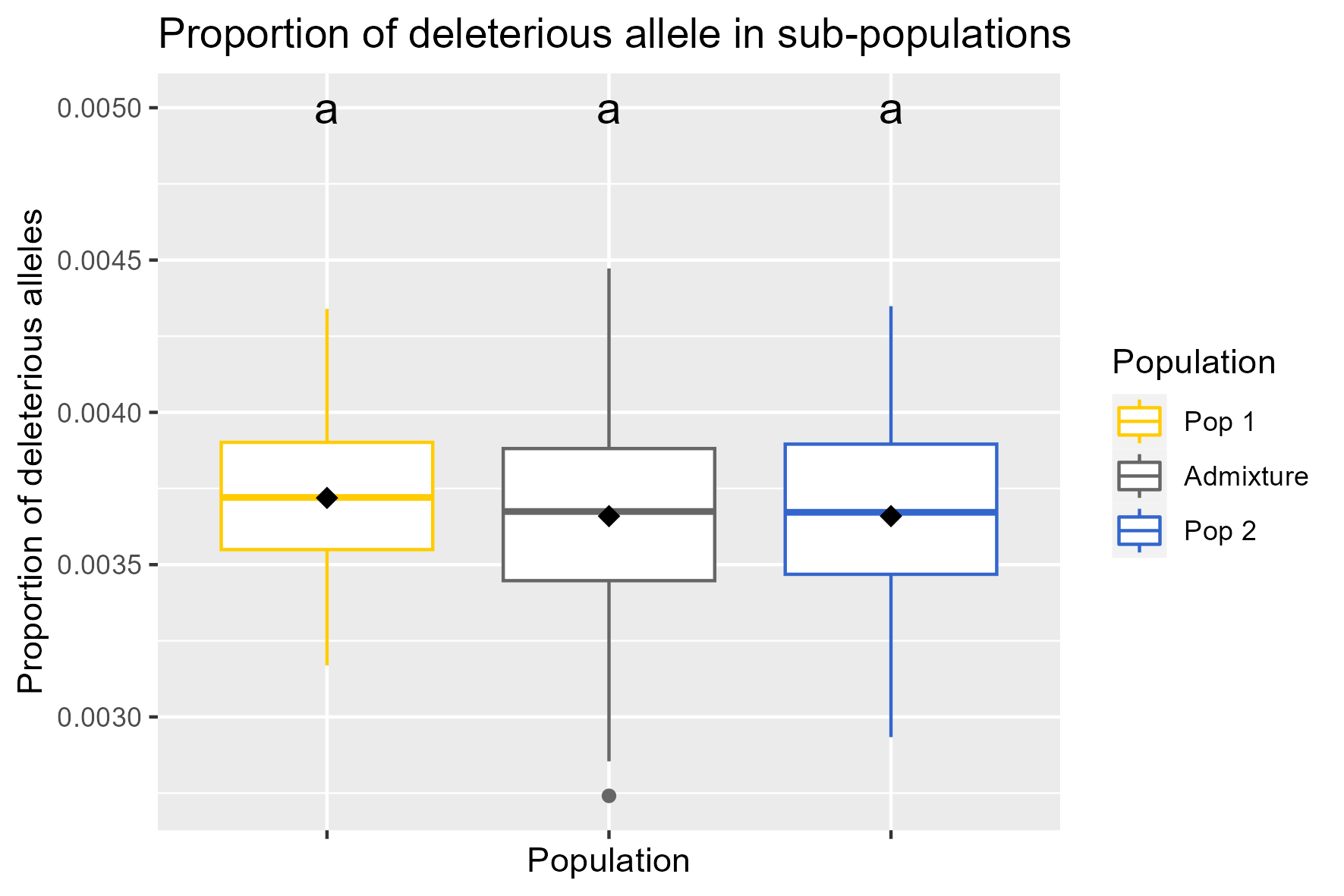


Suppl. Fig. 5: Proportion of deleterious alleles in the subpopulations. Subpopulations were identified in the STRUCTURE analysis. The proportion of deleterious alleles was calculated as the ratio of minor alleles for high impact SNPs over the total number of alleles. The impact of each SNP was predicted by snpEff ® according to position in the genome and an annotated reference sequence. The letters correspond to the result of a Tukey test indicating the significance of the differences observed between subpopulations.


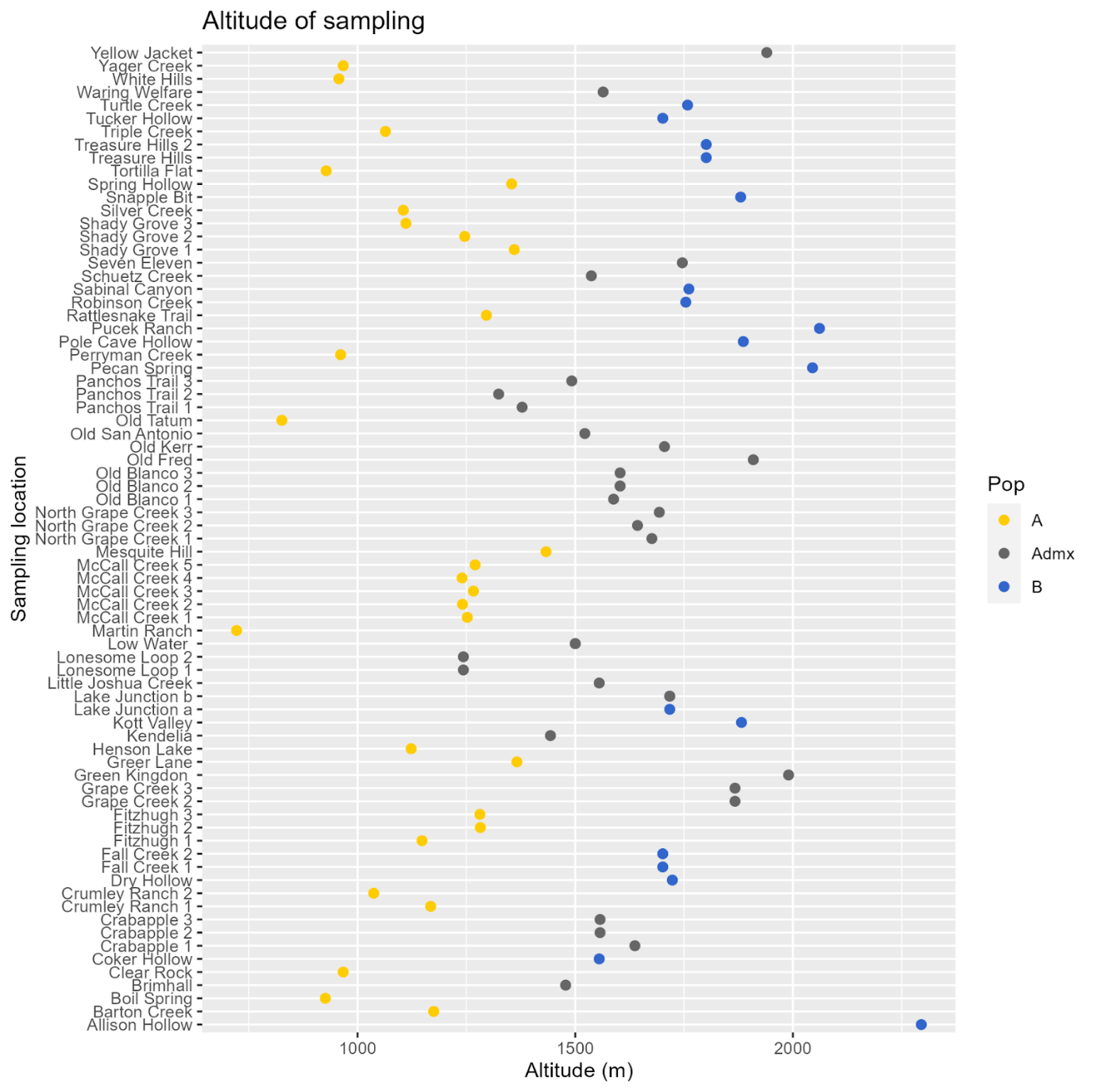


Suppl. Fig. 6: Elevation of the sampling sites (one sampling site per mother plant). Colors indicate the subpopulations identified by STRUCTURE (K=2)


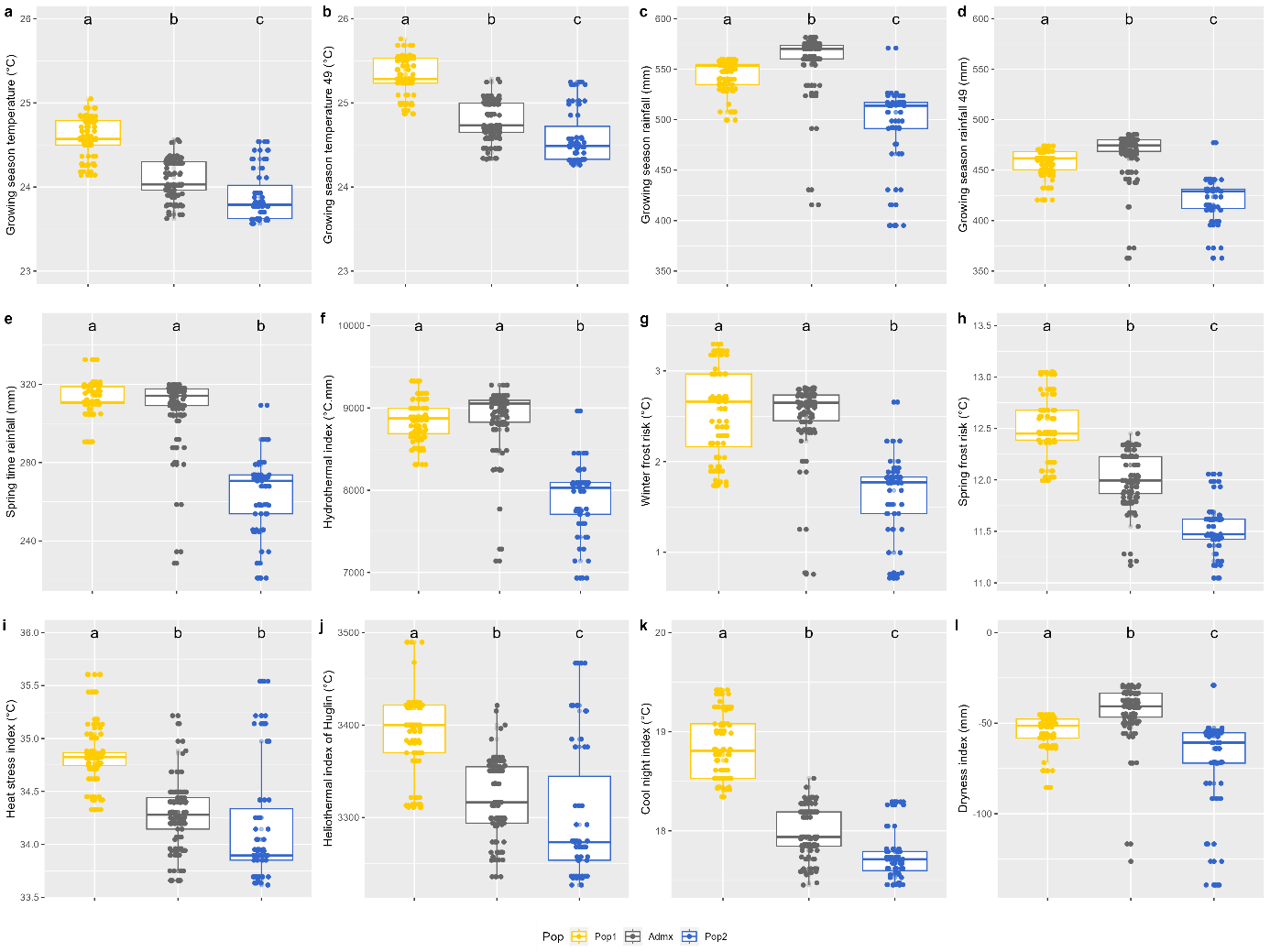


Suppl. Fig. 7: Boxplot of environmental parameters extracted from the TerraClimate platform, including growing season temperature (a), growing season temperature from April to September (b), growing season rainfall (c), growing season rainfall from April to September (d), springtime rainfall (e), hydrothermal index (f), winter frost risk (g), spring frost risk (h), heat stress index (i), Huglin’s heliothermal index (j), cool night index (k) and dryness index (l) for each subpopulation identified by STRUCTURE. The letters indicate the results of a Tukey test (different letters indicate significant differences).


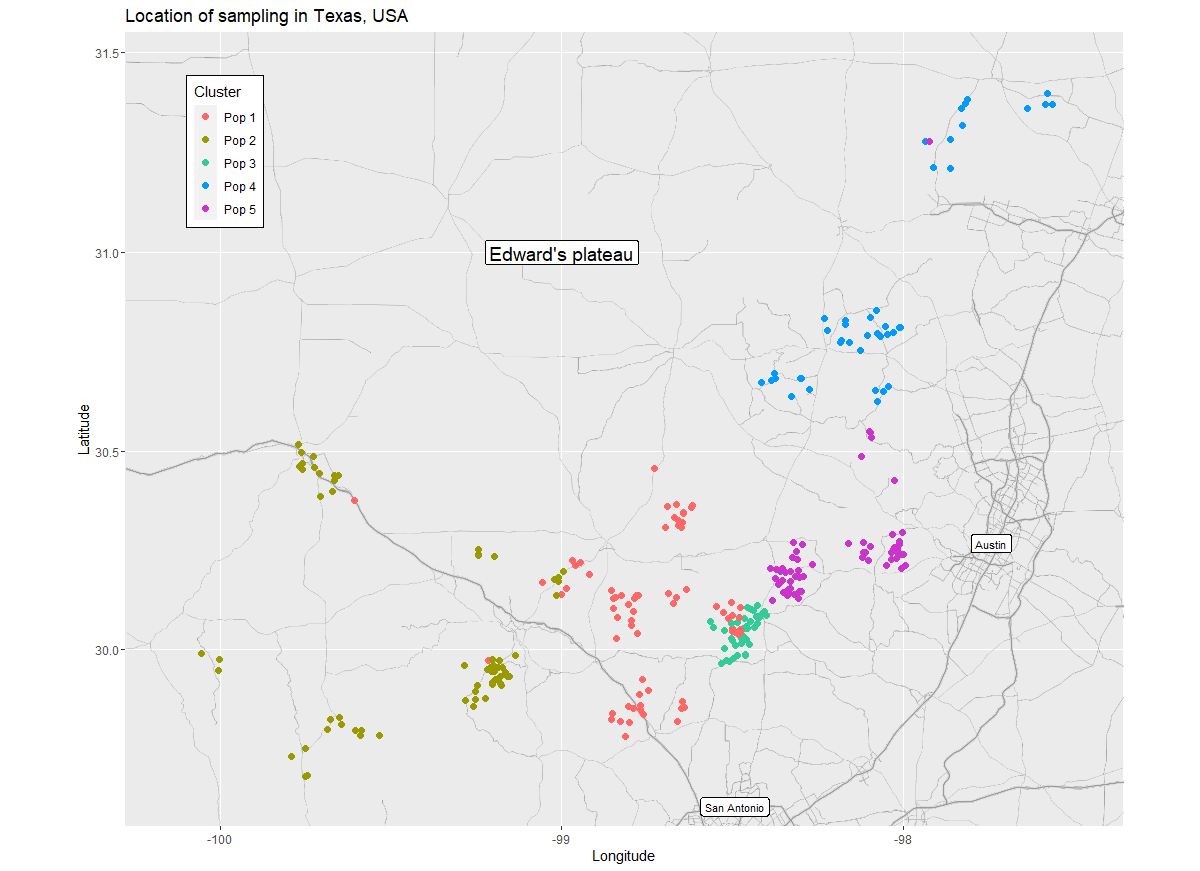


Suppl. Fig. 8: Position of genotypes according to the origin of the mother plant sampled in Texas (each dot corresponds to a genotype from each group identified by the *k*-means clustering method). If the same mother plant gave rise to offspring of several genotypes, the jitter option was used to make it easier to distinguish between genotypes on the map).


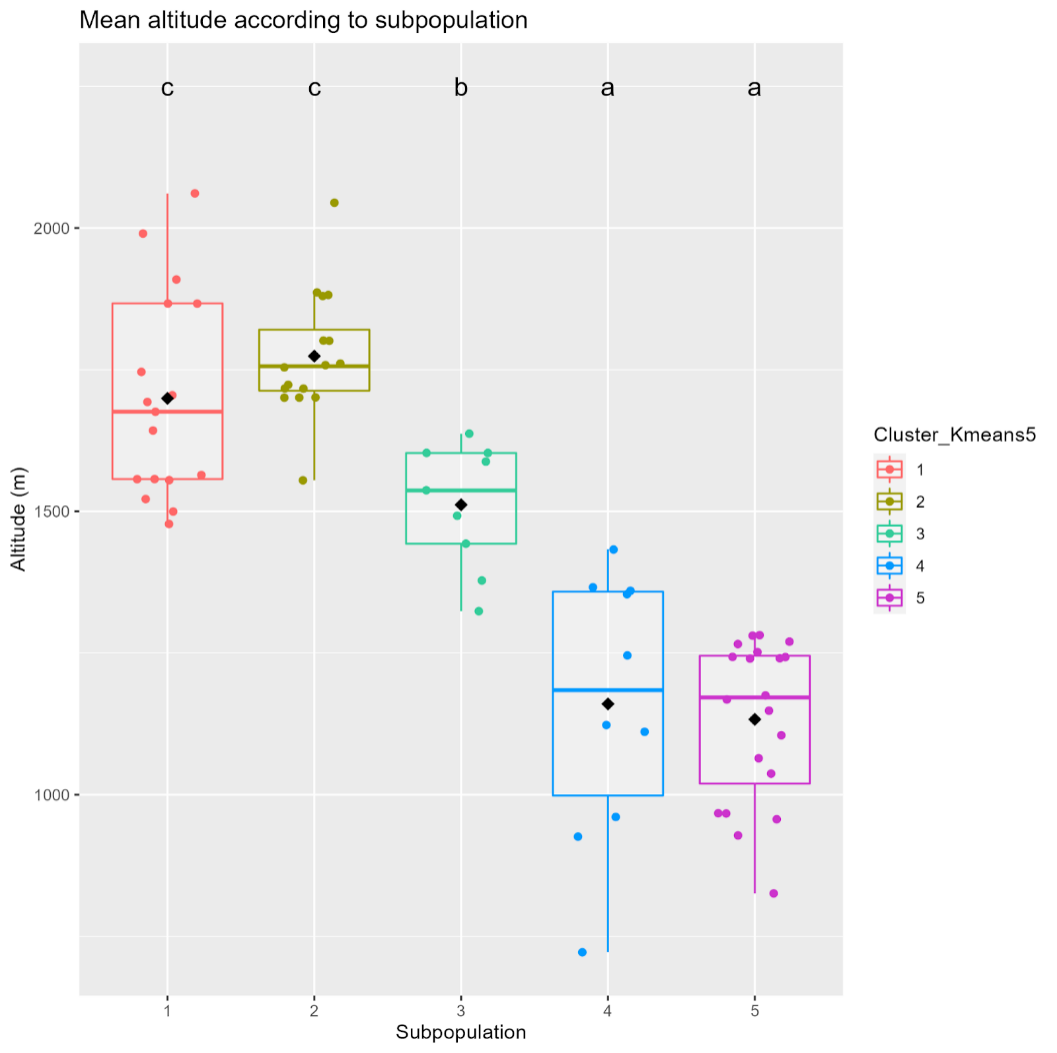


Suppl. Fig. 9: Mean elevation of the subpopulations identified by the *k*-means method (K=5)


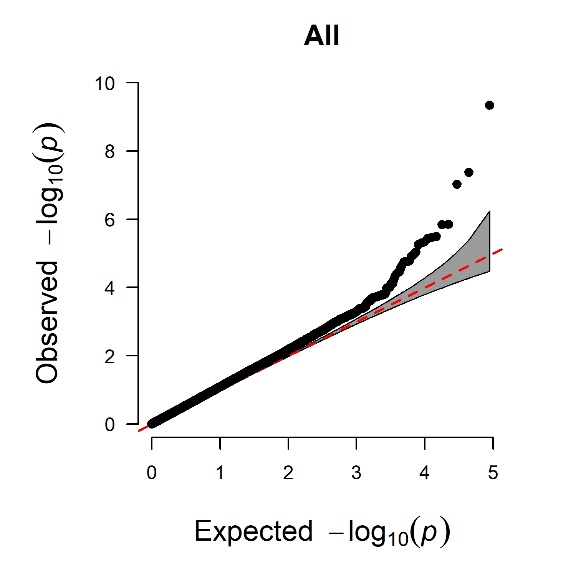


Suppl. Fig. 10: QQ plot, indicating the distribution of theoretical and observed *p*-values in the GEA analysis for ‘elevation’.

**a**

**b**


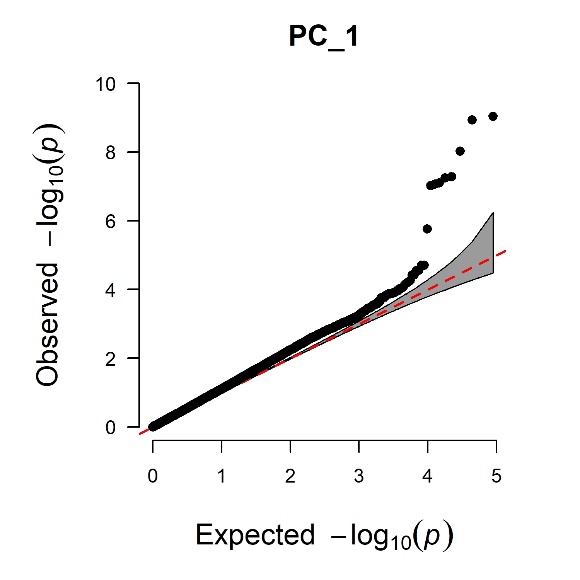

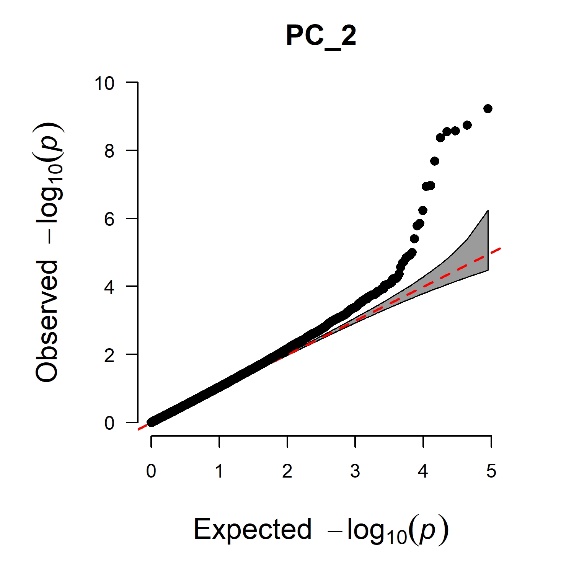


Suppl. Fig. 11: QQ plot, indicating the distribution of theoretical and observed *p*-values in the GEA analysis for PC1 (a) and PC2 (b)


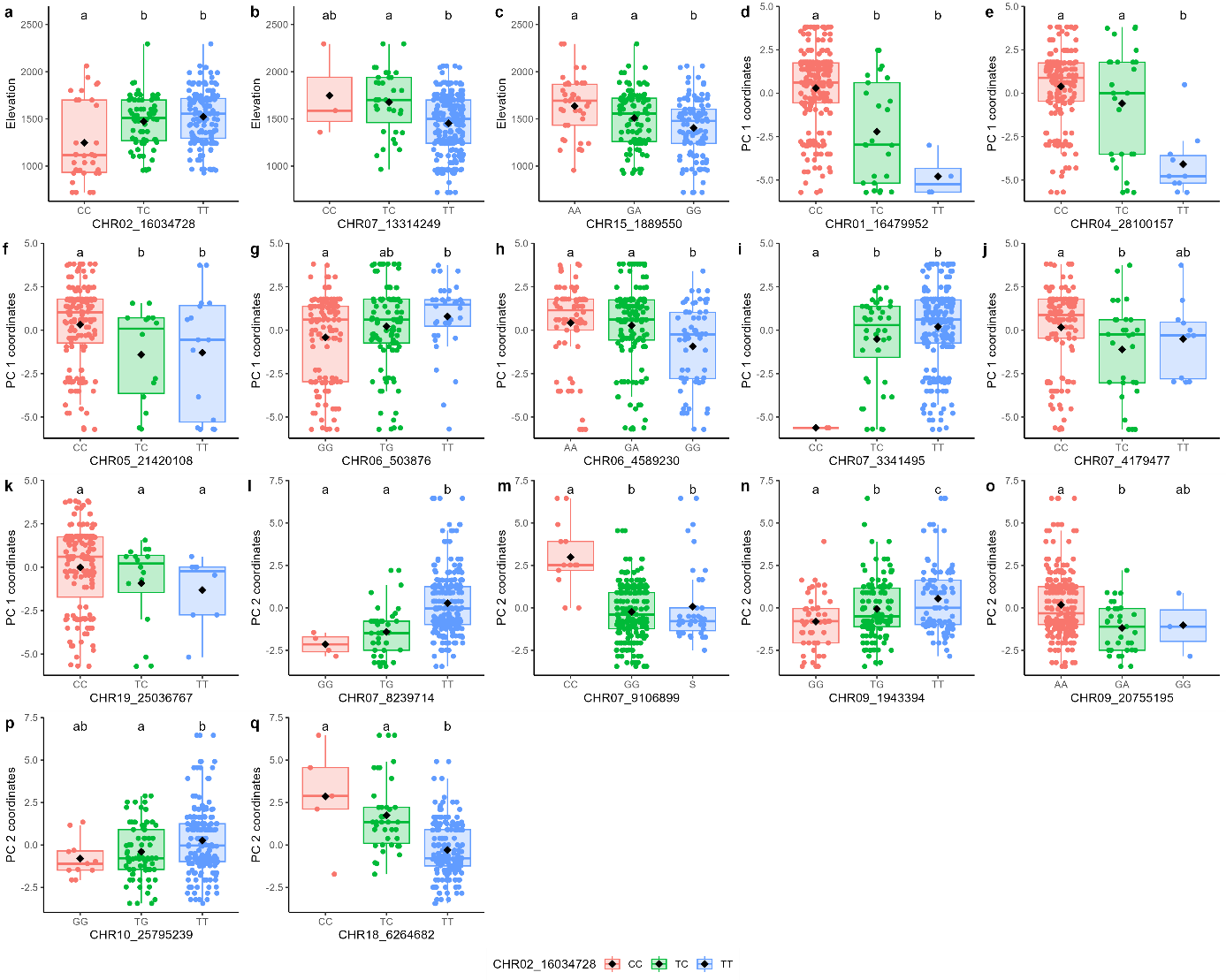


Suppl. Fig. 12: Boxplots of markers effects related to the elevation of sampling (a to c), the first principal component coordinates (PC1, d to k), and the second principal component coordinates (PC2, l to q). All markers detected in GEA analysis have been included.
